# Health risk assessment and ionomic profiling under cadmium, lead, zinc, and manganese stress in *Amaranthus cruentus*

**DOI:** 10.1186/s12870-026-08983-z

**Published:** 2026-05-20

**Authors:** Monika Szabóová, Veronika Lancíková, Jana Kačírová, Andrea Hricová, Veronika Mistríková

**Affiliations:** Institute of Plant Genetics and Biotechnology, Plant Science and Biodiversity Centre, Slovak Academy of Sciences, Nitra, Slovakia

**Keywords:** Human exposure, Metal toxicity, Nutrient content, Pseudocereal, Seed, Soil contamination

## Abstract

**Background:**

Heavy metals (HMs) in agricultural soils pose a serious risk to crop safety and human health. This study evaluated the accumulation of cadmium (Cd), lead (Pb), zinc (Zn), and manganese (Mn) in *Amaranthus cruentus* L. cv. Pribina and integrated ionomic profiling with human health risk assessment to identify tissue-specific responses to metal exposure.

**Results:**

Plants were cultivated in soils supplemented with 15 mg kg^−1^ Cd, 200 mg kg^−1^ Pb, 150 mg kg^−1^ Zn, and 300 mg kg^−1^ Mn up to full maturity. Cadmium accumulation in seeds up to 0.61 mg kg^−1^ exceeded established safety limits, indicating potential health risk to consumers. In contrast, Pb remained below detection limits, and Zn and Mn did not accumulate significantly in harvested seeds. Morphometric traits remained largely unaffected, although seed thickness and surface area decreased under Cd, Mn, and Zn exposure. Histochemical staining revealed Zn localization within protein bodies in seed cross sections. Elemental analysis of Al, Ba, Ca, Cd, Cr, Cu, Fe, K, Mg, Mn, Mo, Na, Ni, Pb, Sr, and Zn revealed stable total nutrient concentrations, but significant alterations in inter-element relationships, suggesting tissue-specific maintenance of nutrient homeostasis. Principal component analysis showed that the first two components explained 81.17% of the total variance (PC1 59.15%, PC2 22.02%), clearly separating plant organs based on their elemental composition. Hierarchical cluster analysis further confirmed the strongest response of roots to HM treatments, emphasizing their primary role in metal uptake and redistribution; the unique behavior of Cd relative to other elements; and the distinct separation of roots treated with Pb.

**Conclusions:**

These findings highlight Cd as the primary risk factor for food safety in contaminated soils and demonstrate that *A. cruentus* cv. Pribina maintains reproductive capacity under elevated metal concentrations, suggesting a notable degree of tolerance to HM stress. This study offers novel insights into the correlation between ionomic plasticity and potential health risks associated with the consumption of edible pseudocereals under environmental contamination.

**Supplementary Information:**

The online version contains supplementary material available at 10.1186/s12870-026-08983-z.

## Introduction

Global food demand is expected to rise by up to 60% by 2050 due to population growth and changing dietary habits, while the availability of productive agricultural land continues to decline because of degradation, urbanization, or pollution [1, 2, 35, 88, 141]. Reusing contaminated soils for crop cultivation or biomass production is therefore a key strategy for sustainable land management [2, 148]. Other critical challenges include minimizing food waste, addressing essential nutrient deficiencies in the food supply, and maintaining food safety [100, 135, 152].

Heavy metal (HM) pollution poses a serious threat to soil health, ecosystems, and human well-being [1, 8, 114]. Cadmium (Cd) is a non-essential, highly mobile element that interferes with plant nutrient uptake and physiology [87, 103, 154] and is readily transferred through the food chain [84]. Chronic Cd exposure causes various health problems in humans, including cancer, osteoporosis, kidney damage, and impaired cardiovascular function [93, 106, 108, 113, 156]. Similarly, lead (Pb) inhibits plant growth and disrupts metabolism [9, 20, 48, 72, 74] while acting as a neurotoxin and carcinogen in humans [10]. In contrast, certain metals such as zinc (Zn) and manganese (Mn) are essential for human health and also for plant growth and development but can become toxic at elevated concentrations [86, 117, 118]. Chronic overexposure to Mn can lead to a neurological disorder similar to Parkinson's disease and additional symptoms, while Mn deficiency is very rare in humans [81]. Conversely, Zn deficiency is one of the most widespread micronutrient malnutrition issues globally, leading to a wide range of health complications [62]. In addition, common cereal crops (e. g. wheat, rice, and maize) often contain insufficient Zn concentrations to meet human requirements [16].

Maintaining the proper homeostatic balance of micro and macroelements is crucial for both plant development and nutritional quality [151], as well as for the safety of edible plant parts for final consumers when considering health-risk assessment. The ionome, defined as the composition of minerals and trace elements in a tissue or organism [153, 162], provides valuable insight into nutrient-metal interactions and plant responses to environmental stress. Plant ionomic profiles are related to various external stressors to which they are exposed [29]. Moreover, ionomics enables simultaneous assessment of multiple elements, helping identify their interactions and providing insight into nutrient-metal relationships under stress conditions. It can reveal coordinated changes, element competition, disruption of element homeostasis, and tissue-specific responses to HM exposure.

*Amaranthus cruentus* L. is a pseudocereal valued for its nutritional and agronomic traits [11, 55]. The balanced amino acid composition, gluten-free nature, and high biomass yield make amaranth suitable for both food and energy applications [4, 129]. Amaranth is a promising candidate for cultivation on marginal and/or HM-contaminated soils. It is also assumed that two Slovak grain amaranth cultivars, Pribina (*A. cruentus* L.) and Zobor (*A. hypochondriacus* x *A. hybridus*), have great potential for phytostabilization of several HMs [61, 75]. Therefore, amaranth, a resilient and easy-to-grow pseudocereal, could meet the current global demand [4].

Despite growing evidence of amaranth's tolerance to HM stress, limited information is available on how this type of contamination alters the ionomic profile across the plant and the corresponding health implications of seed consumption. Therefore, this study aimed to assess the accumulation and distribution of Cd, Pb, Zn, and Mn in *A. cruentus* under long-term HM exposure. We evaluated the potential health risks associated with seed consumption for both children and the adult human population, and characterized the plant's overall ionomic shifts induced by metal presence. We tested the hypothesis that *A. cruentus* limits the transfer of Cd and Pb to seeds, that Zn and Mn soil supplementation increases the total concentration of these elements in seeds to non-toxic, but nutritionally more valuable levels. At the same time, ionomic adjustments triggered by individual metal, reflecting nutrient-metal interactions and adaptive responses, were explored. Our findings provide important insights into the potential of grain amaranth for safe cultivation and phytostabilization in metal-contaminated soils with associated health risks.

## Materials and methods

### Plant material and semi-field experimental set-up

For the analysis, the grain cultivar Pribina (*Amaranthus cruentus* L.) was used, which was previously bred at the home institute by mutation breeding [40, 55]. Plants were cultivated in pots filled with control or contaminated soil and were located at the field trial site in locality Nitra (48°18′24.5″N 18°05′56.5″E), Slovakia, during the growing season from the beginning of May to the end of September 2021 (about 5 months of cultivation until full seed maturity). Climatic data for the study area, including monthly temperature averages and precipitation during the growing season, are provided in the supplementary material (Fig. S1). The plants were grown under natural rain-fed conditions without supplemental irrigation. Soil moisture was not monitored during the experiment. The cultivation used a commercial universal substrate (Agro CS, Slovakia) with the following basic physicochemical properties: pH 6.29, electrical conductivity (EC) 1.66 mS cm^−1^, and nutrient concentrations of N 110 mg kg^−1^, P 515 mg kg^−1^, K 3025 mg kg^−1^ based on dry weight (DW).

At the beginning, when the amaranth seeds were sown, selected HMs (Cd, Pb, Zn, and Mn) were added to the soil in the following forms and concentrations—CdCl_2_ (Sigma-Aldrich, Saint-Louis, USA; 15 mg kg^−1^), Pb(NO_3_)_2_ (Slavus, Slovakia; 200 mg kg^−1^), ZnCl_2_ (Sigma-Aldrich Production GmbH, Switzerland; 150 mg kg^−1^), MnCl_2_ (Slavus, Slovakia; 300 mg kg^−1^), based on previous studies [61, 75]. The control plants were grown in pots without HM addition. After germination, the plantlets were reduced, and a single plant per pot was maintained. At full maturity, the amaranth tissues were collected for further analyses as follows: roots, leaves, inflorescences and seeds. The experiments were performed in three biological replicates.

### Seed weight and morphometric analysis of seeds

The weight of 500 amaranth seeds (g) was calculated as the average of 5 independent measurements for each treatment (control, Cd, Pb, Zn, and Mn). Dried and undamaged seeds were randomly selected for morphometric analysis. Seeds were placed on a glass slide covered with adhesive tape to prevent their displacement and photographed from the dorsal or lateral side using a LEICA MZ10 F stereomicroscope equipped with a LEICA DFC 420 C camera (LEICA Microsystems GmbH, Germany). Seed area was calculated from the dorsal side and seed thickness from the lateral side of at least 130 seeds using ImageJ version 1.43u software (US National Institutes of Health, Bethesda, MD, USA) (Supplementary Fig. S2).

### Dithizone staining of seeds

The dithizone dye was used to localize the tested HMs in amaranth seeds, as it could create a pink to reddish color in their presence [100, 110]. Both non-imbibed and 24-h imbibed whole intact seeds were stained for 90 min. in the dark in a staining solution composed of 50 mg dithizone (Alfa Aesar, MA, USA) dissolved in 75 mL of acetone, 25 mL of distilled water, and 15 drops of glacial acetic acid (Mikrochem Trade, Pezinok, Slovakia). The stained seeds were washed and observed using LEICA MZ10 F stereomicroscope as described above. For detailed microscopic analysis, permanent seed slides were prepared following the procedure of Hricová et al. [56]. Briefly, the paraformaldehyde-fixed and ethanol-dehydrated seed samples were embedded in Paraplast Plus® embedding medium (Sigma-Aldrich, Saint-Louis, USA), and 10 μm thick sections were cut using a rotary microtome CUT 4055 (micro*Tec* LAborgeräte GmbH, Germany). Sections were placed on glass slides, deparaffinized gradually in a xylene and ethanol series until 70% (v/v) ethanol, and stained in the dithizone solution as before. After staining, the sections were washed with distilled water, dehydrated in ascending ethanol and xylene series, embedded in Entellan™ mounting medium (Merck, Life Science spol s.r.o., Slovakia), and dried. Seed slides were observed using a Zeiss Axioplan 2 light microscope equipped with a Sony DXC-S500 digital camera (Carl Zeiss, Jena, Germany).

### Ionomics

Plant samples were prepared for analysis by inductively coupled plasma optical emission spectrophotometry (ICP-OES; Thermo ICAP 7000 Dual, ThermoFisher Scientific, USA) as previously described by Lancíková et al. [75]. In brief, amaranth tissues were washed in deionized water, dried at 50 °C for 48 h, and ground to a fine powder using a TissueLyzer (TissueLyzer II, Qiagen, Hilden, Germany). Each sample (approx. 500 mg) was mineralized in a high-performance microwave digestion system (Ethos UP, Milestone Srl, Sorisole, BG, Italy) in a solution of 5 mL HNO_3_ (TraceSELECT®, Honeywell Fluka, Morris Plains, USA) and 1 mL H_2_O_2_ (30%, for trace analysis, Merck Suprapur®). The samples and the blank were digested according to the manufacturer´s method for ‘dried plant tissue’ to obtain the best results. The following elements were then analyzed, with the limits of detection (LODs) in mg kg^−1^ given in brackets: Al (0.001), Ba (0.004), Ca (0.105), Cd (0.0003), Cr (0.016), Cu (0.003), Fe (0.001), K (0.607), Mg (0.0005), Mn (0.0004), Mo (0.003), Na (0.215), Ni (0.002), Pb (0.008), Sr (0.002), and Zn (0.001). The multi-element standard solution V for ICP-OES (Sigma-Aldrich Production GmbH, Switzerland) was used for the analysis. Half the LOD was used for statistical analysis if the element concentration was below the detection limit. All analytical correlations achieved R^2^ values greater than 0.999, demonstrating high precision in quantification. The accuracy of the analytical method was assessed using certified reference material (ERM CD281). For target elements, the measured concentrations were compared to the certified values, with recoveries ranging from 85.42 to 106.56%, demonstrating that the method provides reliable quantification. Analytical precision was confirmed by replicate analyses with relative standard deviations below 10%.

The representative soil samples (100 g) were collected from the topsoil layers (0–20 cm), dried at 50 °C for 48 h, homogenized, and passed through a 2 mm sieve. Mineralization was performed as described above for amaranth tissues using a digestion system modified to 8 mL HNO_3_ and 2 mL H_2_O_2_. Subsequent elemental quantification via ICP-OES analysis specifically targeted the concentration of supplemented HMs in both control and treated substrates (Supplementary Table S1).

### Assessment of soil pollution, bioconcentration, and translocation factors

The degree of soil pollution for each HM in the pot experiment was assessed at harvest using the contamination factor (CF). The CF values were calculated using Eq. (1), as described by Rezapour et al. [107]:

1 $$\mathrm{C}\mathrm{F}=\frac{Cmetal}{Ccontrol}$$

*C*_metal_ and *C*_control_ (mg kg^−1^; based on DW) represent the concentrations of each HM in the contaminated and control soil samples, respectively.

The bioconcentration factor (BCF), which indicates the phytoextraction potential of plants, and the root-shoot translocation factor (TF) of selected HMs were calculated according to Zheng et al. [164] and Coakley et al. [18] as following Eqs. (2) and (3), respectively:

2 $$\mathrm{B}\mathrm{C}\mathrm{F}=\frac{Cplant}{Csoil}$$

3 $$\mathrm{T}\mathrm{F}=\frac{Cshoot}{Croot}$$

*C*_plant_, *C*_soil_, *C*_shoot_, and *C*_root_ denote the concentration of each HM (mg kg^−1^ DW) in the total plant biomass, soil, shoot biomass (comprising leaves, inflorescences, and seeds), and roots, respectively.

### Assessment of human health risk

In this study, the potential non-carcinogenic risk (NCR) and carcinogenic risk (CR) to human health from consumption of HM-contaminated amaranth seeds were evaluated using a previously published methodology, as described below. The average daily intake (ADI; mg kg^−1^ day^−1^) of a given metal from amaranth seeds consumption was calculated according to Zhang et al. [161] following Eq. (4):

4 $$\mathrm{A}\mathrm{D}\mathrm{I}=\frac{c\times IR\times EF\times ED}{BW\times A}\times {10}^{-6}$$

where *C* is the metal concentration in amaranth seeds (mg kg^−1^), *IR* is the ingestion rate of seeds (mg day^−1^), *EF* is the exposure frequency (350 days year^−1^), *ED* is the exposure duration (year), *BW* is the average body weight (kg), and *AT* is the average exposure time (days). Unlike staple cereals such as wheat, amaranth seeds are generally consumed only as a dietary supplement in smaller quantities, with intake levels similar to those of quinoa or buckwheat, other members of the pseudocereal family [45, 73]. Applying the same baseline *IR* values for wheat grains (75,000 and 150,000 mg day^−1^ for children and adults, respectively) as adapted by Zhang et al. [161] will not ensure an accurate risk assessment that reflects the amaranth consumption pattern. To address this discrepancy, the *IR* values for amaranth seeds were reduced by a factor of three for both children and adults, to 25,000 and 50,000 mg day^−1^, respectively (Table 1).

**Table 1:** Input parameters used in this study to assess non-carcinogenic and carcinogenic risks for children and adults

Parameters and units	Children	Adults
Ingestion rate of amaranth seeds (*IR*, mg day^−1^)	25,000	50,000
Exposure frequency (*EF*, days year^−1^)	350	350
Exposure duration (*ED*, year)	6	24
Average body weight (*BW*, kg)	29	63
Average exposure time (*AT*, days)	*ED* × 365, for NCR 76.6 × 365, for CR	same as for children same as for children

Input parameters were adapted from Zhang et al. [161], except for *IR* values, which were adjusted to reflect the specific intake levels of amaranth seeds. Abbreviations: carcinogenic risk (CR), non-carcinogenic risk (NCR)

The non-carcinogenic risk (NCR) was assessed using Eq. (5) for the calculation of the target hazard quotient (THQ) as proposed by the United States Environmental Protection Agency (US [137],US [138]:

5 $$\mathrm{T}\mathrm{H}\mathrm{Q}=\frac{ADI}{RfD}$$

*ADI* (mg kg^−1^ day^−1^) represents the average daily intake of a single metal by oral ingestion, and R*f*D (mg kg^−1^ day^−1^) is the reference dose for the human population with no appreciable risk of adverse effects during life. The R*f*D for the intake of Cd, Pb, and Zn is reported as 0.001, 0.0035, and 0.3, respectively [23, 64, 140, 161], and 0.14 for Mn according to the Integrated Risk Information System [140].

The carcinogenic risk (CR), an index that estimates the probability that a person will develop cancer during their lifetime if exposed to a certain dose of a potential carcinogen, was calculated in this study for only two carcinogenic metals (Cd and Pb) as follows Eq. 6 [140, 161](US:

6 $$\mathrm{C}\mathrm{R}=ADI\times SF$$

SF (mg kg^−1^ day^−1^) indicates the slope factor, the associated cancer risk, for each metal over the consumption route. According to Cheng et al. [23], *SF* is over 6.1 and 0.0085 for Cd and Pb, respectively. No *SF* values are given for Zn and Mn as they are not known carcinogens [140].

### Statistical analysis

Data processing and statistical analyses were performed using GraphPad Prism 9.4.1 software. Statistical differences in the accumulation of analyzed elements between control plants and plants from HM-contaminated soils were assessed using one-way ANOVA followed by Benjamini–Hochberg false discovery rate (FDR) correction. Effect sizes were calculated as Cohen's *f* based on the ANOVA results. A correlation network was used to visualize overall ionomic interactions based on Pearson correlation coefficients [46]. Only strong correlations (r > 0.7 and *p* < 0.05) were included in the network to reduce the noise. The resulting networks were visualized using Cytoscape v.3.9.1 software [112]. Given the limited sample size, these patterns are interpreted as exploratory and indicative of potential interactions rather than definitive relationships. In addition, one-way ANOVA followed by Tukey’s HSD test was used to determine significant differences in seed weight and morphometric parameters at *p* ≤ 0.05. Principal component analysis (PCA) was performed to predict the relationships among variables, including plant tissue, HM treatment, and micronutrient composition. The PCA was used as an explanatory multivariate approach based on the correlation structure of the dataset. Ionomic responses in amaranth tissues to HM stress were further examined using hierarchical cluster analysis (HCA) in R (version 4.1.1), and a heat map was created using the “pheatmap” package [71]. The HCA was performed using the average linkage method and the Euclidean distance, and element concentrations were scaled prior to analysis. All samples were analyzed in three biological replicates.

## Results

### Distribution of heavy metals in soil and amaranth tissues

The final concentrations of supplemented Cd, Pb, Zn, and Mn in the top soils sampled at the harvest are presented in Supplementary Table S1. According to these data, Cd- and Pb-supplemented soils were found to be extremely polluted, with CF values of 108.35 ± 12.77 and 81.91 ± 22.12, respectively. Soils supplemented with Zn and Mn showed a moderate degree of contamination, with CF values of 2.78 ± 0.48 and 2.20 ± 0.76, respectively.

The accumulation and distribution patterns of these metals in the below- and above-ground tissues of *A. cruentus* are summarized in Table 2. Cadmium accumulated predominantly in the roots, with concentrations decreasing in the order: root > leaf > inflorescence > seed. Although the TF was relatively low (0.39), Cd concentrations in leaves and inflorescences were significantly high. Moreover, among the tested metals, only Cd in the seeds was significantly increased compared to control, with a value of 0.61 mg kg^−1^. The BCF for Cd was 0.48, indicating moderate soil uptake.

**Table 2:** Bioconcentration and translocation of selected heavy metals in amaranth tissues

Metals	Tissue	Concentration (mg kg^−1^ DW)	BCF	TF
Cd	Root	32.72 ± 8.06*		
	Leaf	7.56 ± 1.14*		
	Inflorescence	3.76 ± 1.94*		
	Seed	0.61 ± 0.65*		
			0.48 ± 0.08*	0.39 ± 0.15
Pb	Root	893.65 ± 362.24*		
	Leaf	< LOD		
	Inflorescence	< LOD		
	Seed	< LOD		
			0.78 ± 0.14*	0.00 ± 0.00
Zn	Root	179.66 ± 63.14		
	Leaf	126.21 ± 59.84*		
	Inflorescence	74.71 ± 10.63*		
	Seed	66.60 ± 6.35		
			1.40 ± 0.24*	1.52 ± 0.15*
Mn	Root	13.35 ± 5.34		
	Leaf	5.50 ± 1.85		
	Inflorescence	2.55 ± 1.04		
	Seed	1.40 ± 0.83		
			0.01 ± 0.01	0.90 ± 0.73

Data are means of three biological replicates ± standard deviations. Statistically significant differences compared to control plant tissues, determined with a t-test at *p* < 0.05, are marked with *

*Abbreviations*: bioconcentration factor (BCF), dry weight (DW), limit of detection (LOD), translocation factor (TF)

In contrast to Cd, there was only minimal translocation of Pb. Lead accumulated in relatively high amounts in the roots, while concentrations in the leaves, developing inflorescences, and seeds remained below the detection limit (LOD < 0.008 mg kg^−1^). Despite a comparatively high BCF (0.78), Pb was almost completely retained in the root system, with negligible translocation to the above-ground parts (TF = 0.00).

Zinc was mainly stored in roots and leaves, with concentrations gradually decreasing in inflorescences and seeds. Notably, Zn content in leaves and inflorescences was significantly lower under Zn supplementation compared to control plants (Table 2). Among the analyzed metals, Zn was the only element with both BCF and TF values exceeding one (1.40 and 1.52, respectively), indicating effective uptake from soil and active translocation within the plant. However, both factors were significantly lower compared to control, revealing Zn exclusion strategy. Considering effective translocation of Zn to aerial plant parts, the lower concentration in leaves and inflorescences compared to control was probably caused by regulatory mechanisms that limit Zn accumulation in metabolically sensitive tissues to prevent toxicity.

Despite Mn soil supplementation, both below- and above-ground plant organ concentrations remained similar to control. Nevertheless, Mn exhibited a pattern of accumulation similar to that of Cd, with the highest levels detected in the roots and decreasing levels in the above-ground parts. The BCF for Mn was very low (0.01), while the TF almost reached one (0.90), suggesting efficient internal redistribution within the plant despite minimal uptake from the soil.

### Human health risk associated with the intake of heavy metals from amaranth seeds

The estimated ADI values for Cd, Pb, Zn, and Mn in the amaranth seeds for children and adults are shown in Table 3. Based on these ADI values, the THQ and CR were calculated to assess the potential non-carcinogenic and carcinogenic health risks associated with consumption of HM-contaminated seeds via ingestion (Table 3). According to standard US EPA [137, 139] guidelines, a THQ < 1 suggests a negligible NCR, while a THQ > 1 indicates a potential adverse non-carcinogenic health effect. For carcinogenic risk, CR values between 1 × 10^–6^ and 10^–4^ are considered to be within an acceptable risk range, whereas values exceeding 10^–4^ denote a significant health hazard.

**Table 3:** Assessment of average daily intake and health risks for children and adults from consumption of amaranth seeds grown on heavy metal-contaminated soils

Metals	Population	ADI for NCR	ADI for CR	THQ	CR
Cd	Children	5.06 × 10^–4^	3.96 × 10^–5^	0.51	2.42 × 10^–4^
	Adults	4.68 × 10^–4^	1.46 × 10^–4^	0.47	8.90 × 10^–4^
Pb	Children	3.31 × 10^–6^	2.59 × 10^–7^	9.45 × 10^–4^	2.20 × 10^–9^
	Adults	3.04 × 10^–6^	9.54 × 10^–7^	8.70 × 10^–4^	8.10 × 10^–8^
Zn	Children	5.51 × 10^–2^	-	0.18	-
	Adults	5.09 × 10^–2^	-	0.17	-
Mn	Children	1.16 × 10^–3^	-	0.01	-
	*Adults*	*1.07* × *10*^*–3*^	*-*	*0.01*	*-*

*Abbreviations*: *ADI* average daily intake, *CR* carcinogenic risk, *NCR* non-carcinogenic risk, *THQ* target hazard quotient

For Cd-contaminated amaranth seeds, we estimated THQ values of 0.51 for children and 0.47 for adults, both within the tolerable limits, indicating a low risk for non-cancer diseases. However, CR values exceeded the acceptable limits for both age groups and were higher in adults than in children. For Pb, the THQ values for children and adults (9.5 × 10^–4^ and 8.70 × 10^–4^, respectively) and the CR values for adults (8.10 × 10^–8^) were below risk thresholds, while CR values for children (2.20 × 10^–9^) were within the acceptable limit, indicating no health risk from Pb exposure via seed consumption. For Zn and Mn, THQ ranged from 0.01 to 0.18, remaining well below 1, suggesting that consumption of seeds from Zn- and Mn-supplemented soils is unlikely to pose a health risk.

### Seed weight and morphometric analysis

To evaluate the potential effects of excessive HMs in soil, their bioavailability and uptake by amaranth plants on seed parameters, we specifically analyzed seed weight, surface area, and thickness under Cd, Pb, Zn, and Mn treatments (Table 4; Fig. S2). Seed weight did not differ significantly between HM-treated and control plants. However, morphometric analysis revealed a significant reduction in both seed surface area and thickness after Cd, Zn, and Mn application. In contrast, seeds from plants grown on Pb-contaminated soil showed no significant differences in any measured parameter compared to control.

**Table 4:** Seed weight and morphometric parameters under different heavy metal treatments

**Treatments**	**Seed weight (g)**	**Seed surface area (mm**^**2**^**)**	**Seed thickness (mm)**
Control	0.43 ± 0.02	1.22 ± 0.15	0.93 ± 0.06
Cd	0.42 ± 0.01	1.18 ± 0.18*	0.88 ± 0.06*
Pb	0.44 ± 0.01	1.20 ± 0.14	0.91 ± 0.06
Zn	0.44 ± 0.02	1.14 ± 0.17*	0.91 ± 0.05*
Mn	0.44 ± 0.02	1.18 ± 0.13*	0.90 ± 0.04*

Data are means ± standard deviations of measurements of amaranth seeds from different pot treatments (control, Cd, Pb, Zn, and Mn). Statistically significant differences compared to control seeds at *p* < 0.05 are marked with *

### Histochemical localization of heavy metals in amaranth seeds

**Fig. 1:**
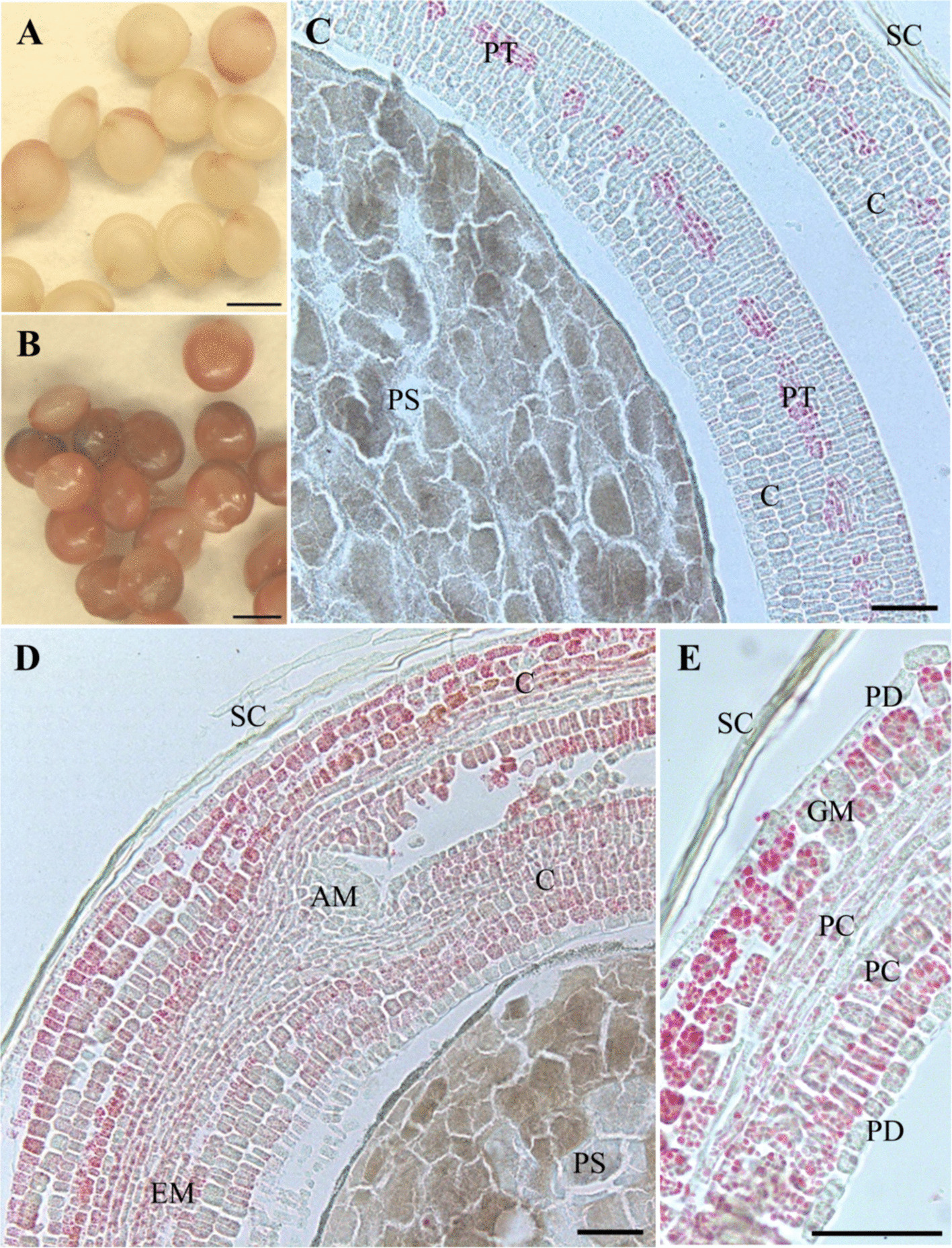
Representative image of dithizone staining of tested heavy metals in non-imbibed (**A**) and 24-h imbibed (**B**) seeds of *A. cruentus* cv. Pribina, and seed cross-sections (C-E). Parts of two cotyledons (**C**), apical meristem (AM), embryo axis (EM), seed coat (SC), starchy perisperm (PS), and differentiated seed tissues, such as provascular tissue (PT), protoderm (PD), ground meristem (GM), and procambium (PC) are seen in C-E. Part of the cotyledon is shown at higher magnification in (E). Protein bodies are observed as discrete pink spots within cells. Bar = 1 mm (A, B); 50 μm (C – E)

Dithizone staining was performed to localize the tested HMs within the seeds (Fig. 1). Non-imbibed intact seeds (Fig. 1A) showed only slight coloring sporadically on the periphery of the seed coat, whereas water absorption (Fig. 1B) allowed the staining solution to penetrate the seed, resulting in overall intense pink-purple staining. Staining of permanent seed slides revealed that all metals were specifically localized in protein bodies, appearing as discrete pink deposits inside cells within the seed embryo. We observed two distinct localization patterns for protein bodies: centralized in the cotyledons, probably within the provascular tissue, and in the protoderm (Fig. 1C), or spread throughout the whole embryo, especially within the ground meristem and procambium (Fig. 1D, E). No staining was detected in the starchy perisperm or seed coat. The staining pattern was the same across all groups, including seeds from control and HM-treated plants. While dithizone-based screening remained unable to differentiate less abundant metals such as Cd, Pb, or Mn, the prevalence of Zn in the seeds was clearly revealed. The ICP-OES seed ionomic analysis provided the necessary quantitative data to support histological observations, highlighting the importance of multi-method approaches (Table 2).

### Ionomic response of amaranth tissue to heavy metal stress

**Fig. 2:**
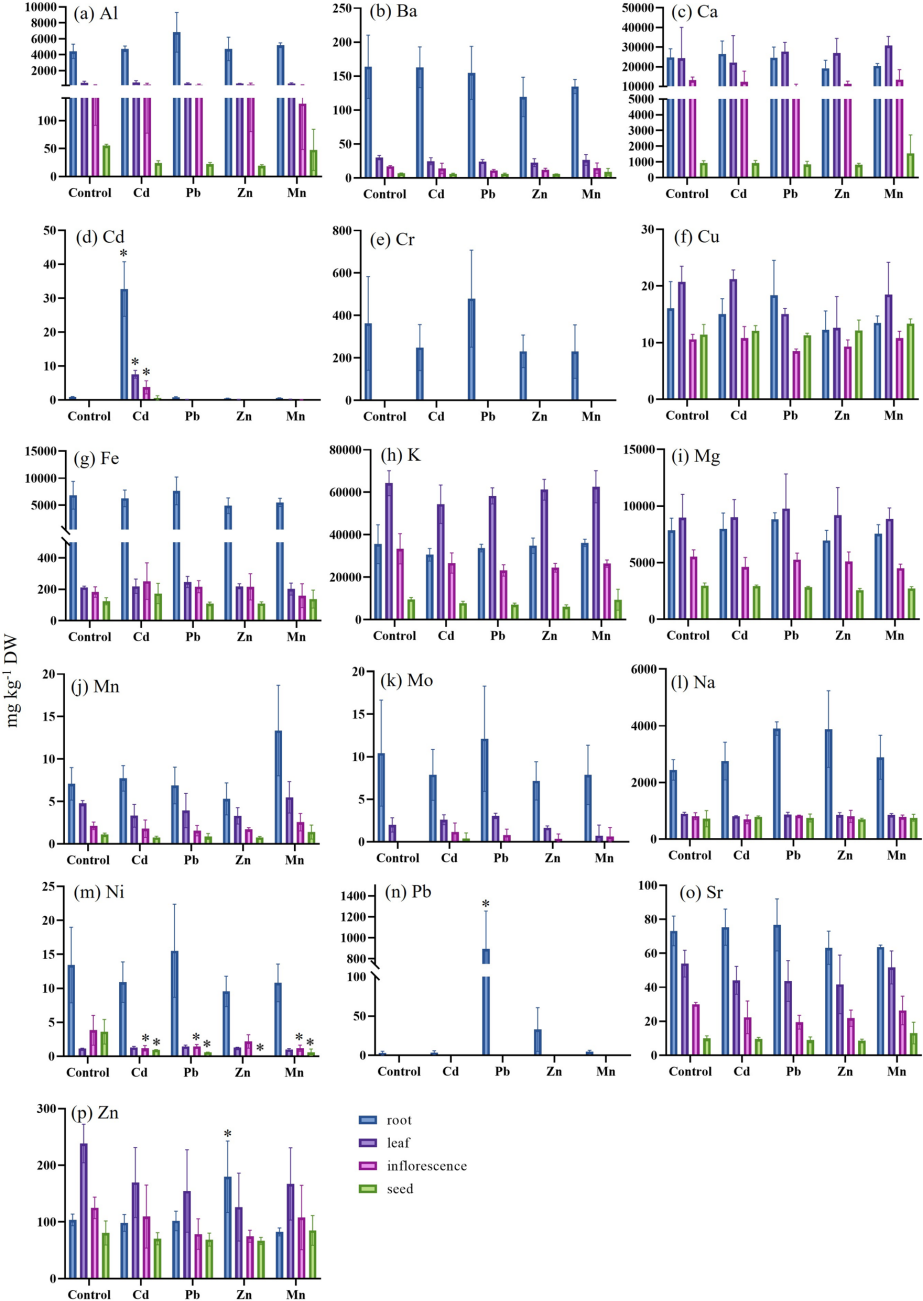
Concentrations (mg kg.^−1^ DW) of Al (**a**), Ba (**b**), Ca (**c**), Cd (**d**), Cr (**e**), Cu (**f**), Fe (**g**), K (**h**), Mg (**i**), Mn (**j**), Mo (**k**), Na (**l**), Ni (**m**), Pb (**n**), Sr (**o**) and Zn (**p**) in amaranth tissues treated with different heavy metals. Statistically significant differences among treatments were determined using one-way ANOVA followed by FDR correction, with significant effects marked with *. Data are means ± standard deviations (*n* = 3). Abbreviation: dry weight (DW)

In this work, we attempted to determine the ionomic responses in amaranth plants exposed to individual Cd, Pb, Zn, and Mn stresses. The concentrations of sixteen elements determined by ICP-OES across roots, leaves, inflorescences, and seeds of HM-treated plants are shown in Fig. 2. Detailed statistical results, including effect sizes (Cohen's *f*) and FDR-adjusted *p*-values derived from one-way ANOVA, are provided in Supplementary Table S2.

In general, the concentrations of Ba, Cd, Cr, Fe, Mn, Mo, Na, and Ni were higher in roots of amaranth than in the above-ground parts of plants, while K, Mg, and Zn mainly accumulated in the leaves (Fig. 2). Obvious significant changes in the concentration of applied HMs within the amaranth tissues have already been described above (Table 2). Additionally, Cd exposure appeared to reduce K uptake, although this effect was not statistically significant (Fig. 2). In Zn-treated plants, Ca, Cu, and Mg concentrations in the roots decreased slightly compared to control. Manganese supplementation reduced Zn content in roots, although this effect was not statistically significant after FDR correction. However, a moderate effect size was observed (Supplementary Table S2). Conversely, Zn-treated plants exhibited the lowest Mn content in the roots (Fig. 2).

In amaranth leaves, we observed only minor changes in elemental composition after HMs application (Fig. 2). Although the presence of Cd and Pb tended to reduce K and Zn concentrations, these changes were not statistically significant. In addition, Cd treatment decreased Mn content. Excessive Pb content in the soil negatively affected Cu levels in both leaves and developing inflorescences. In seeds, Al concentration decreasedafter the addition of Cd, Pb, and Zn, while K content was reduced after the application of Pb and Zn compared to the control plants.

As expected, statistically significant treatment effects after FDR correction were primarily observed for Cd concentrations across tissues. This effect may reflect strong accumulation under Cd exposure. In addition, significant effects were detected for Ni in inflorescences and seeds. Overall, although only a limited number of effects were statistically significant, several elements showed moderate to large effect sizes, indicating potential treatment-related trends (Supplementary Table S2).

**Fig. 3:**
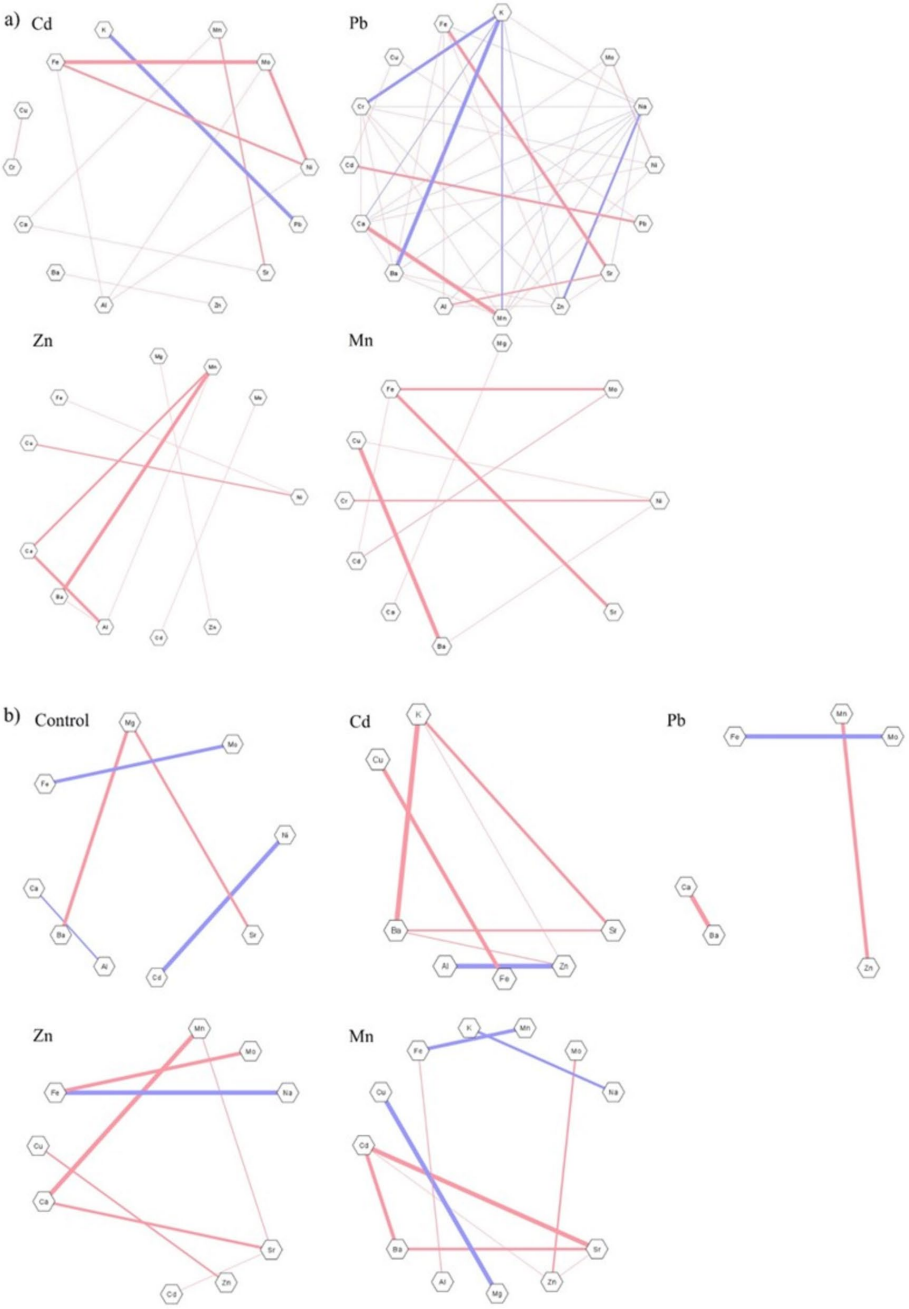
Ionomic networks in roots (**a**) and leaves (**b**) of amaranth plants grown in the control conditions and in the presence of Cd, Pb, Zn, and Mn, generated by Cytoscape v.3.9.1 software. The correlations between the elements in the treatments were evaluated using Pearson´s correlation analysis in GraphPad Prism 9.4.1 software at a significance level of *p* < 0.05 and r > 0.7. Red lines indicate a positive correlation, blue lines indicate a negative correlation; the thicker the line, the stronger the correlation. Only statistically significant correlations are presented

**Fig. 4:**
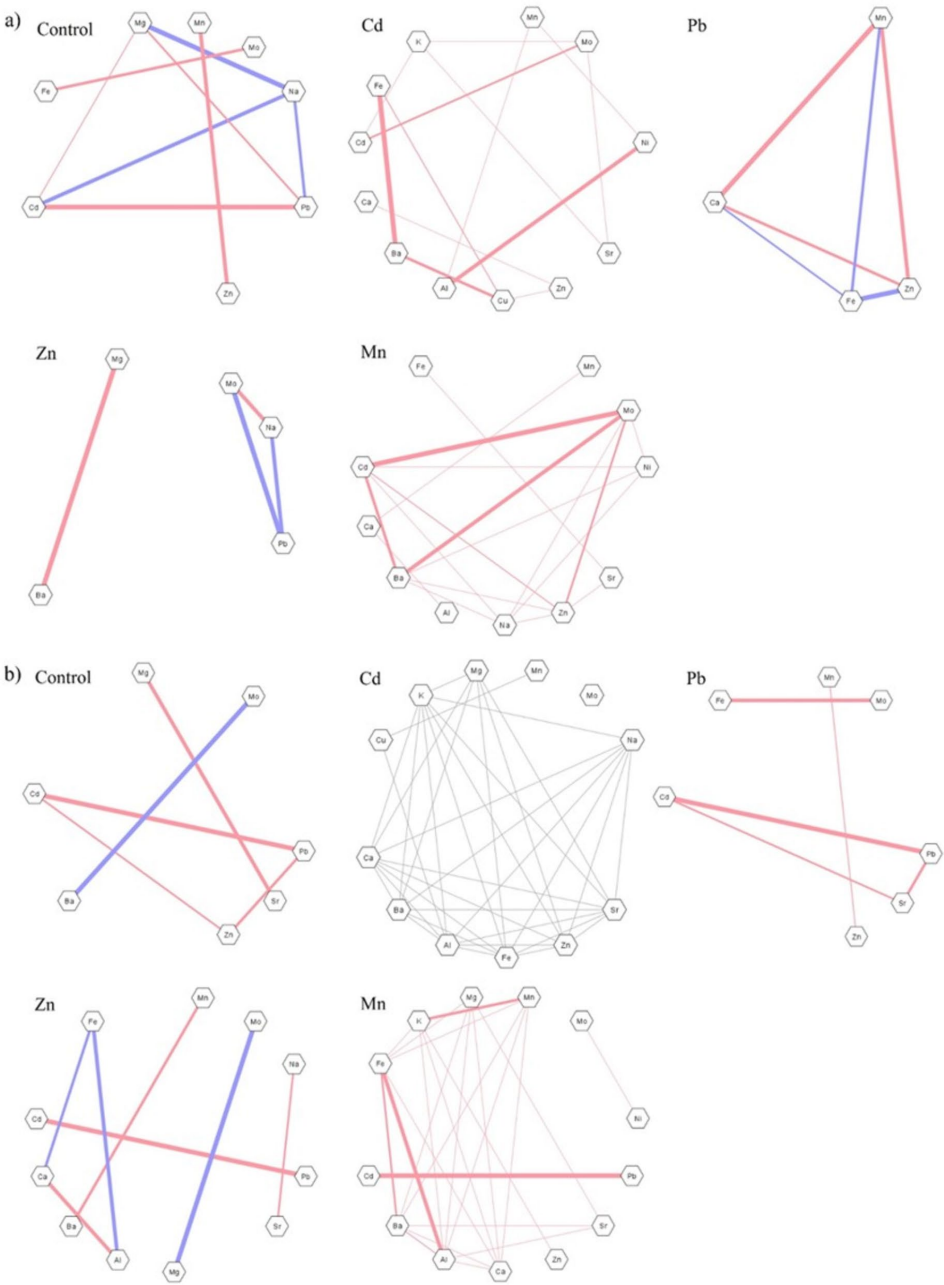
Ionomic networks in inflorescences (**a**) and seeds (**b**) of amaranth plants grown in the control conditions and the presence of Cd, Pb, Zn, and Mn generated by Cytoscape v.3.9.1 software. The correlations between the elements in the treatments were evaluated using Pearson´s correlation analysis in GraphPad Prism 9.4.1 software at a significance level of p < 0.05 and r > 0.7. Red lines indicate a positive correlation, blue lines indicate a negative correlation; the thicker the line, the stronger the correlation. Only statistically significant correlations are presented. In Cd-treated amaranth seeds, gray lines indicate that only positive correlations were observed

Correlation matrices for each plant tissue (root, leaf, inflorescence, and seed) under all experimental groups (control, Cd, Pb, Zn, and Mn) are shown in the Supplementary Material (Tables S3-S6). The corresponding ionomic network visualizations are presented in Figs. 3 and 4. The summary table of network parameters is presented in Table S7. While the total concentrations of most micro- and macroelements were not significantly altered by different HM (Fig. 2), the elemental correlation patterns in amaranth tissues were clearly affected. This suggests tissue-specific modifications in element coordination, potentially linked with nutrient homeostasis under HM stress. Such patterns may reflect changes in nutrient regulation, although causal relationships cannot be established from correlation analysis alone. In the presented networks (Fig. 3, Fig. 4) only strong correlations were included; nevertheless, these results remain exploratory given the limited number of replications.

In the roots of plants grown under control conditions, no significant correlations were observed among the detected elements (Table S3). Several significant positive correlations occurred under Cd and Pb stress, including Al/Fe, Ba/Zn, and Mo/Ni (Fig. 3, Table S3). In addition, a consistent significant positive correlation was observed between Ca and Mn in response to excess Cd, Pb, and Zn.

The roots of amaranth plants grown in Pb-contaminated soil showed the most complex correlation pattern, with 25 pairs of significant positive and 14 significant negative element correlations (Fig. 3, Table S3). Lead appeared to influence the uptake of Cu and Fe, both of which showed, though statistically not significant, increases in concentration (Fig. 2). In contrast, the roots of plants cultivated in Zn- and Mn-treated soils showed significant positive correlations for Cu/Ni and Cd/Mo (Fig. 3, Table S3). No significant negative correlations were observed among the elements in the roots under Zn or Mn excess. Although Zn positively correlated with Al, Ba, Ca, and Sr, these relationships were not statistically significant.

Several positive (Ba/Mg, Mg/Sr) and negative correlations (Al/Ca, Fe/Mo, Cd/Ni) were observed among the elements detected in the leaf tissue of plants grown under control conditions (Fig. 3, Table S4). The ionomic profiles of plants exposed to Cd and Pb stress revealed significant positive correlations, including Ba/K, Ba/Sr, Ba/Zn, Cu/Fe, K/Sr, K/Zn and Ba/Ca, Mn/Zn, respectively. Lead stress generated a significant negative correlation of Fe/Mo in the leaves, and Cd stress only had a negative effect on the correlation of Al/Zn. In response to Zn stress, a significant positive correlation was observed between Zn and Cu, as well as Ca/Mn, Ca/Sr, Cd/Sr, Mn/Sr, and Fe/Mo. A significant negative correlation was observed between Fe and Na in leaves under Zn stress. In addition, Mn treatment led to a significant negative correlation between Mg/Cu, Mn/Fe, and Mo/K. On the other hand, a significant positive correlation was observed between Al/Fe, Ba/Cd, Ba/Sr, Cd/Sr, Cd/Zn, Mo/Zn and Zn/Sr in the leaves of Mn-treated plants.

When evaluating the ionome in the developing inflorescence, several positive (Cd/Mn, Cd/Pb, Fe/Mo, Mg/Pb, Mn/Zn) and negative correlations (Cd/Na, Mg/Na, Na/Pb) were observed among analyzed elements in plants grown under control conditions (Fig. 4, Table S5). However, treatments with Cd and Mn showed the greatest number of correlations between the elements, with 13 and 18 statistically significant correlation pairs, respectively. Briefly, Cd was significantly correlated with K and Mo, while Mn showed a significant positive correlation with Ca. Excessive addition of Zn and Pb to the soil showed few statistically significant correlations.

Amaranth seeds from control plants showed only four positive correlations (Cd/Pb, Cd/Zn, Mg/Sr, Pb/Zn) and one negative correlation (Ba/Mo). We observed a complex pattern of correlations under Cd stress in amaranth seeds, involving 35 pairs of significant correlations among the analyzed elements (Fig. 4, Table S6). However, we could not find any direct correlation between Cd and other analyzed elements. In the case of Pb, only positive significant correlations were observed between Fe/Mo, Mn/Zn, Pb/Sr, Pb/Cd, and Sr/Cd. The stress induced by excessive Zn supply led to a significant positive correlation between Ba/Mn, Ca/Al, Fe/Al, Cd/Pb, Na/Sr, and to a negative correlation including Al/Fe, Fe/Ca, and Mo/Mg. In seeds from Mn-treated plants, 24 significant positive correlation pairs were found, while Mn correlated with Al, Ba, Ca, F, and K.

**Fig. 5:**
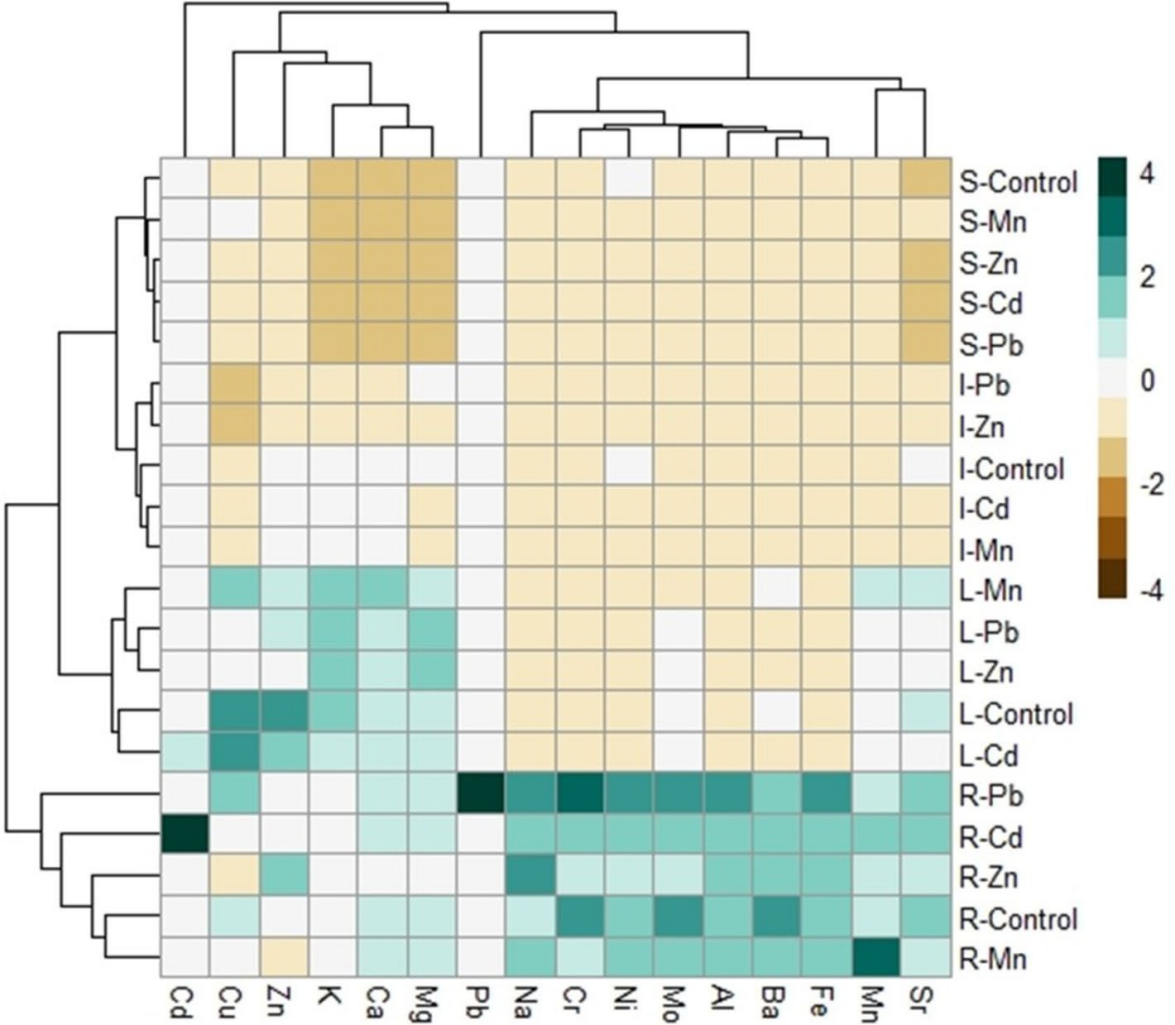
Hierarchical cluster analysis (HCA) of the ionome in the roots (R), leaves (L), inflorescences (I) and seeds (S) of amaranths treated with Cd, Pb, Zn, Mn and untreated control group. The HCA was generated in R version 4.1.1 software and a heat map was created using the “pheatmap” package. The HCA used the average method and the Euclidean distance between elements. The concentrations of the individual elements were scaled before HCA. Brown stands for a low concentration and green for a high concentration of the elements

**Fig. 6:**
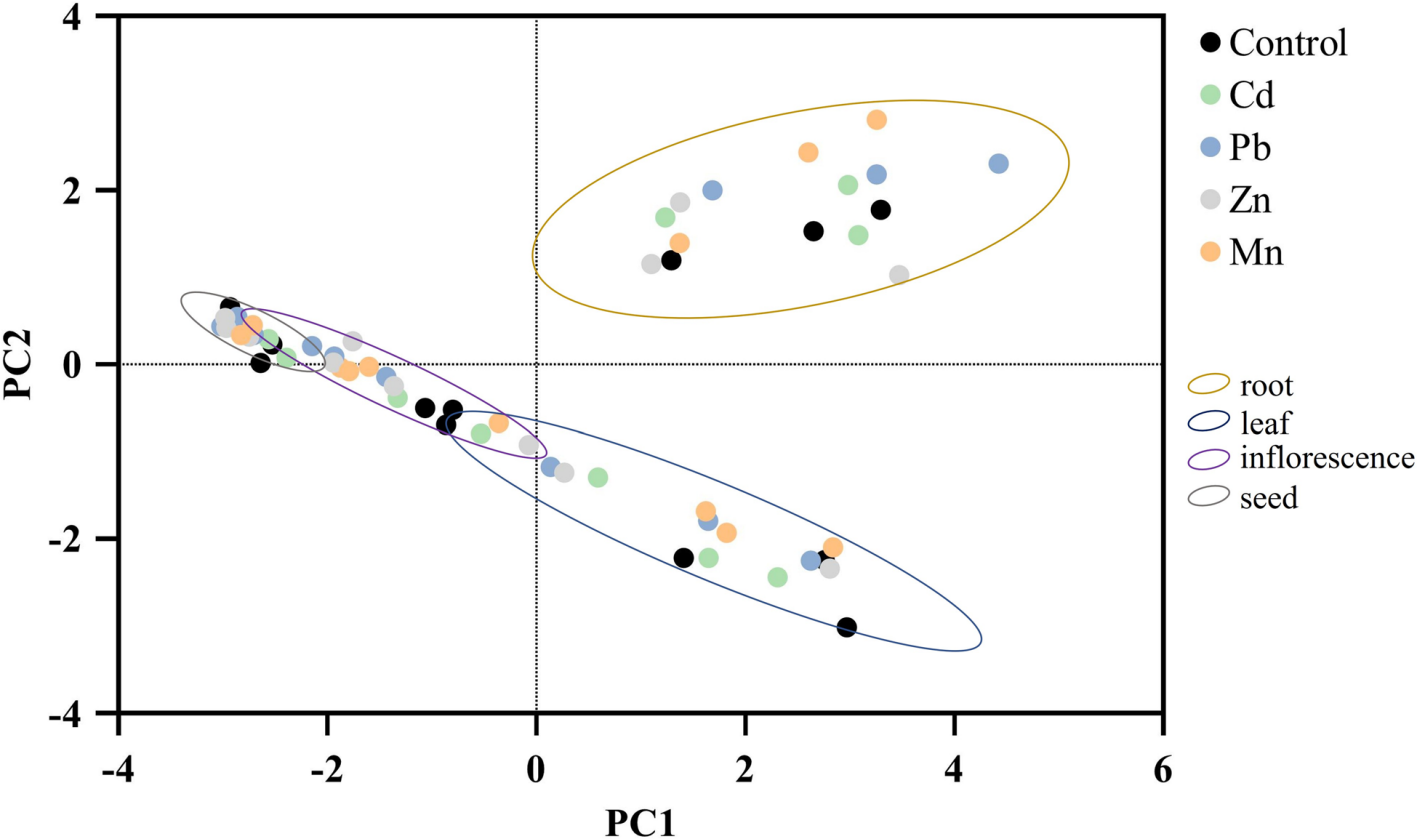
Principal component analysis** (**PCA) of elemental distribution in amaranth plant tissues (seed, root, leaf, and inflorescence). The loading plot (top) shows the contribution of individual elements to the first two principal components (PC1 and PC2). Elements with similar vector orientations are positively correlated, whereas those in opposite directions are negatively correlated. The score plot (bottom) illustrates the distribution of samples by organ type based on ionomic profiles

The ionomic response of amaranth to Cd, Pb, Zn, and Mn treatment during the growth period was further characterized by HCA (Fig. 5) and PCA (Fig. 6). In the HCA, elements and plant tissues were clustered separately to identify homogeneous subgroups, with results visualized as a two-dimensional heat map accompanied by dendrograms (Fig. 5). Based on ionomic profiles, amaranth tissues (roots, leaves, inflorescences and seeds) were grouped into four distinct clusters, reflecting tissue-specific elemental distribution. Notably, the distinct separation of root samples under Pb treatment supported Pb-induced changes in micro- and macroelements, consistent with findings from correlation analysis and elemental distribution data (Fig. 2).

Elemental clustering revealed two major groups: cluster 1 included only Cd, while cluster 2 comprised the remaining elements studied (Fig. 5). This classification highlighted the unique behavior of Cd in comparison to other elements and supported its distinct physiological impact on ionomic profiles in amaranth tissues.

The association between ionomic responses to stress from four different HM treatments in amaranth tissues is illustrated by PCA in Fig. 6. Principal component 1 (PC1) accounted for 59.15% of the total variance, while PC2 explains 22.02%. The proportion of variance explained by each component is presented in Supplementary Fig. S3. PC1 clearly separated reproductive tissues (inflorescences, seeds) from vegetative tissues (roots, leaves), indicating major differences in overall elemental composition. Roots and leaves were further distinguished by PC2, reflecting differences in the accumulation of specific elements. Overall, the PCA revealed a clear separation among plant tissues, although seeds and inflorescences contributed only minimally to the overall variability. In leaf tissues, variability was predominantly explained by PC1, whereas PC2 primarily influenced root tissues. Root tissues were closely associated with Fe, Na, and Mn, while leaf tissues showed associations with Ca, Cu, K, Mg, and Zn (Fig. 6). These tissue-specific elemental associations were consistent with the subgroup classifications identified in the HCA dendrograms.

## Discussion

Both metals, Cd and Pb, are widely recognized as highly hazardous and non-degradable HMs that pose significant risks to human and animal health, as well as the environment, even at low concentrations [43, 78, 126]. In contrast, Zn and Mn function as essential micronutrients vital for biological health. Deficiency in these micronutrients can lead to malnutrition, while toxicity occurs only at very high concentrations [6, 77, 89]. Investigating these elements concurrently enables a comparative assessment of uptake, translocation, and accumulation behavior in *A. cruentus* under different HM supplementations designed to mimic contaminated environments. The concentrations of HMs selected for this study were adapted from previous hydroponic studies [61, 75] and adjusted for pot experiments to ensure measurable responses in amaranth. Although these concentrations exceeded levels typically found in natural or contaminated soils, they were intentionally applied to induce high-stress conditions under a controlled framework. It should be noted that, since this study utilized newly spiked soils with freshly added HMs, the observed uptake and accumulation rates likely overestimate the plant's responses compared to natural field conditions [145]. The limitations of the experiments with newly spiked soils include the potentially high bioavailability and lower stability of the added HMs, compared to native field-collected soils with a consistent degree of HM contamination. Additionally, it is important to note that while our study did not focus on measuring precipitation, the water regime is a critical factor that can also influence soil metal mobility and concentrations [49, 159]. Conversely, interpreting data from field-contaminated soils may present several challenges, including the simultaneous presence of multiple metals, varying chemical forms of the deposited HMs, and the heterogeneous levels of HM contamination across the site [91].

### Distribution of heavy metals in amaranth tissues

The ability of plants to accumulate Cd varies considerably, not only among species, but also among cultivars within a single species [24, 75, 79]. In the present study, Cd distribution in amaranth organs was as follows: root > leaf > inflorescence > seed. Similar Cd accumulation patterns were also observed in other Amaranthaceae members, such as spinach leaves [65] and sugar beet roots [123], as well as in unrelated vegetables, including cabbage [31, 42], onion [127], and carrot [31, 65]. The distribution pattern observed in our study differs from that reported by Adekunle et al. [3], who found the highest Cd concentration in *A. cruentus* stems, followed by roots, suggesting cultivar-specific Cd accumulation.

In contrast, Pb accumulation in *A. cruentus* occurred exclusively in the roots, with concentrations in leaves, inflorescences, and seeds below the detection limit. This contradicts previous studies reporting significant Pb levels in the plant's edible parts. For instance, Aguilar et al. [5] detected Pb concentrations of 30.20 mg kg^−1^ and 35.56 mg kg^−1^ in dried seeds of *A. cruentus* and *A. hypochondriacus*, respectively. Sulaiman et al. [120] also reported a Pb concentration of 0.84 mg kg^−1^ in roots and 0.05 mg kg^−1^ in leaves of *A. gangeticus*. However, it is important to note that leafy vegetables tend to accumulate higher levels of HMs compared to fruits, seeds, and grains [92]. Liu et al. [78] investigated Pb accumulation in leafy amaranth *A. tricolor* under combined soil and atmospheric contamination. The authors observed predominant Pb accumulation in the roots and significant levels in the leaves. Under our experimental conditions, Pb accumulation in amaranth plants was almost entirely restricted to the roots, as soil exposure was the only source of Pb. Our statement is confirmed by air quality monitoring data, according to which no air HM pollution was detected in experimental locality in Nitra [119]. These findings support the hypothesis that Pb accumulation in leaves is mainly influenced by atmospheric deposition, whereas soil contamination typically results in Pb retention in roots [78, 163]. Roots of many plants, such as beans, peas, wheat, and rice, absorb the majority of Pb, with only a non-significant amount transferred to the aerial parts [136]. Moreover, similar root-specific Pb accumulation was observed in *Sinapis alba* grown in Pb-contaminated soils [142]. Additionally, strong retention of Pb in plant roots is considered an important tolerance mechanism that effectively limits its mobility to above-ground parts and could be accompanied by several morpho-anatomical changes [78].

Elevated Zn levels in soil are phytotoxic and can alter normal physiological and biochemical activities in plants, thereby reducing their growth and development [68, 89]. Plants can activate genes encoding transcription factors, enzymes, channels, and transporters to prevent excessive Zn absorption and accumulation [26]. In this study, amaranth roots and leaves were identified as the main organs of Zn accumulation, consistent with previous findings [61, 131]. However, increased soil Zn concentrations resulted in decreased Zn levels in leaves and inflorescences, while Zn levels in roots and seeds remained unchanged compared to control. Similar results were observed in cereals, where soil-applied Zn had little effect on grain Zn concentrations [157],Cakmak et al., 2018). Although Zn uptake from the soil was efficient in amaranth, its transport to the above-ground parts of the plant was limited to avoid potential toxicity. Plants must often tightly regulate Zn translocation to leaves and reproductive tissues and tend to accumulate Zn in roots, where it can be sequestered safely in vacuoles or bound to cell wall components [76, 122].

Amaranth plants responded to Mn treatment similarly to Cd and Zn exposure, with the highest Mn concentration in the roots, though the difference was not statistically significant. Overall, Mn treatment did not significantly alter its levels in any plant organ. These results are consistent with previous findings indicating a high degree of Mn tolerance in *A. cruentus* cv. Pribina [61]. As reported, the ability of plants to tolerate elevated Mn levels is associated with activation of the antioxidant system, regulation of Mn transport and homeostasis, and compartmentalization of Mn into subcellular compartments [77].

### Bioaccumulation potential and translocation factor of heavy metals in amaranth

The contamination factor (CF) is commonly used to evaluate soil contamination risk, with four categories: low (CF < 1), moderate (1 ≤ CF < 3), considerable l (3 ≤ C F < 6), and very high (CF ≥ 6) [52]. At the end of our experiment, Cd and Pb levels in the topsoil were significantly elevated compared to controls, indicating a very high degree of contamination (Table S1). The estimated levels of Zn and Mn in soil reflected a moderate degree of soil contamination, but the concentrations were not significantly higher than those of the control soil. Both BCF and TF were calculated to assess the phytoextraction potential of amaranth plants. Bioaccumulation, defined as the ability of plants to absorb and retain contaminants from soil, is a crucial parameter for evaluating human health risks associated with the consumption of contaminated plants [97, 120]. However, BCF values are strongly influenced by soil chemical properties and HM speciation [124, 155].

In the present study, BCF values for *A. cruentus* were 0.48 (Cd), 0.78 (Pb), 1.40 (Zn), and 0.01 (Mn). These results suggest moderate Cd and Pb uptake efficiency, high Zn uptake potential, and negligible Mn accumulation. Notably, Pb exhibited a relatively high BCF, despite being almost entirely restricted to the roots. Zhou et al. [166] reported a higher BCF for Cd (3.15) in *A. viridis*, highlighting species- and environment-dependent variability. Similarly, Sun et al. [121] determined a Cd BCF of 1.3 in *A. hypochondriacus*, while Huang et al. [59] reported BCF < 1 for both the roots and leaves of *A. spinosus*. High levels of soil Pb are known to impair biochemical and physiological processes, including grain development and nutritional quality in cereals [10]. Our results are consistent with those of Ogunkule et al. [97] and Zhou et al. [166], who also reported Pb BCF < 1 in amaranth species. For Zn, the calculated BCF (1.40) indicates efficient uptake and mobility in the plant. However, Zn supplementation reduced its concentrations in leaves and inflorescences, while Zn levels in roots and seeds remained stable. Higher Zn BCFs (2.93–9.39) were reported in lettuce, radish, bok choy, and tomato plants treated with Zn nanoparticles and Zn metal ions [70], suggesting that uptake efficiency may depend strongly on the Zn form. In contrast, minimal bioaccumulation of Mn (0.01) in *A. cruentus* confirms the plant's ability to limit and tightly regulate its uptake, preventing excess accumulation under the tested conditions. As reported previously by White and Neugebauer [149], the Mn uptake capacity of root cells is often determined by the plant's nutritional status for other elements.

Translocation factor, defined as the ratio of metal concentration in the shoots to that in the roots, is used to evaluate the mobility of metals within the plant. A TF > 1 indicates preferential translocation to the shoots, while TF < 1 typically reflects retention in roots. In *A. cruentus*, the highest TFs were observed for Zn (1.52) and Mn (0.90), suggesting efficient Zn mobility and moderate Mn transport. Despite limited Mn translocation from the soil, once Mn was absorbed, it was readily translocated to the above-ground organs. Comparable Zn translocation (TF > 1) was reported in *Brassica juncea* [22]. Although Zn translocation from root to shoot involves multiple regulatory steps, further research is required to clarify the molecular mechanisms governing Zn partitioning into seeds [41]. Cadmium showed a TF of 0.39, indicating limited redistribution. This agrees with previous findings in hydroponically grown *A. cruentus* under similar Cd concentration [75] and in *A. gangeticus*, where TF values were 0.63 (root-to-leaf) and only 0.019 (root-to-seed) [120]. Lead exhibited negligible translocation to above-ground parts, consistent with previous observations in *A. cruentus, A. hypochondriacus x A. hybridus* [61] and *A. dubius* [85]. Immobility of Pb is attributed to its limited apoplastic transport compared to Cd, which is more redistributed to edible organs such as leaves and seeds [10, 105]. Overall, despite Pb showing the higher BCF than Cd, Pb remained largely confined to the roots, whereas Cd was effectively translocated to leaves, inflorescences, and seeds. These contrasting behaviors highlight fundamental differences in the mobility and potential dietary risks of these metals in amaranth.

### Health risk associated with heavy metal intake from amaranth seeds

Approximately 90% of the human HMs intake comes from consuming vegetables and fruits, while the remaining 10% is attributed to dermal contact and inhalation of contaminated dust [64, 69]. Thus, food safety related to cereals, pseudocereals, and vegetables represents a serious global concern [1, 82, 84, 98]. Pseudocereals such as quinoa (*Chenopodium quinoa* Willd), amaranth (*Amaranthus* sp.), and buckwheat (*Fagopyrum* sp.) are of particular importance due to their high nutritional and nutraceutical value and suitability for people suffering from coeliac disease, wheat allergies and gluten intolerance [4, 45, 56, 83, 125, 129]. However, direct consumption of seeds from contaminated areas may result in increased accumulation of toxic elements in humans and animals. Health risk assessment of dietary metals commonly relies on THQ and CR. A THQ < 1 indicates a low NCR, whereas THQ value > 1 suggests potential adverse health effects. Regarding the possible carcinogenic effects, CR values < 1 × 10^–6^ are considered insignificant, values in the range of 1 × 10^–6^ to 1 × 10^–4^ fall within acceptable range, and CR > 1 × 10^–4^ is regarded as unacceptable [98, 120, 137].

In this study, Cd concentrations in amaranth seeds (0.61 mg kg^−1^ DW; Table 2) exceeded the FAO/WHO [37] maximum allowable limit of 0.1–0.2 mg kg^−1^ established for cereal grains and quinoa. Although THQ for Cd remained below the tolerable limit and indicated a low risk of development of non-cancer diseases, the calculated CR values exceeded recommended standards, indicating a significant cancer risk. The estimated CR was 3.68 times higher in adults compared with children, likely reflecting the cumulative longer lifetime exposure assumed for adult intake. Lead, another non-essential toxicant, is known to induce phytotoxicity and severe health effects to humans when accumulated beyond safety thresholds [78]. In our study, the measured Pb concentrations in the seeds were not only below the maximum permissible limits (0.1–0.2 mg kg^−1^) set by the FAO/WHO (2021) but also below the concentration thresholds that would indicate NCR or CR. Similar findings were reported by Nag and Cummins [90], who observed low to moderate risks from Pb exposure through the consumption of potato, carrot, and leafy vegetables. Consistently, Sulaiman et al. [120] reported only low NCR for Pb in leafy amaranths, whereas Cd in *A. gangeticus* was linked to a low NCR but elevated CR. Comparable patterns were also reported in wheat grown in wastewater-irrigated soils, where Cd posed negligible NCR but moderate CR, again with higher risks for adults than for children [107].

While Zn and Mn are essential micronutrients for plants, animals, and humans, excessive accumulation can be associated with toxicity affecting nervous, cardiovascular, or reproductive systems [28, 111]. To ensure a balance between nutritional adequacy and safety, the European Food Safety Authority (EFSA) established safe intake ranges of 2.9–12.7 mg day^−1^ for Zn and 2–8 mg day^−1^ for Mn [32, 33]. In the present study, the calculated ADI of both elements in children and adults remained below the recommended limits (Table 3), and thus did not even reach the tolerable upper intake level of up to 25 mg day^−1^ for Zn in adults established by the Scientific Committee on Food [109]. Correspondingly, the THQ values for both elements ranged from 0.01 to 0.18, indicating that consumption of these seeds poses no health risk. Crucially, the seeds retained a Zn and Mn nutritional profile comparable to control samples, suggesting that *A. cruentus* can maintain beneficial micronutrient balance without posing a toxicological risk under Zn or Mn soil supplementation. As highlighted by the recent studies of Ullah et al. [132, 133], monitoring the nutritional richness of food and the level of environmental contamination is necessary to ensure health security.

### Seed weight and morphometric analysis

Our results demonstrated that HM stress had pronounced effects on seed morphometric traits in *A. cruentus*, particularly under Cd, Zn, and Mn contamination. Although no significant differences were observed between seeds from Pb-contaminated soils and controls in this study, Ndlovu et al. [94] reported that Pb exposure significantly increased seed weight in *Corchorus olitorius*.

In our study, plants grown in Cd-contaminated soils produced seeds with the most impaired parameters, including seed weight and thickness. Such alternations could negatively impact germination, yield, and overall seed quality, while simultaneously elevating potential health risks for consumers, as discussed above. Further research is required to clarify whether these changes result from Cd-induced physiological stress during growth, direct Cd accumulation in the seeds, or a combination of both, and whether these responses are species- or cultivar-specific. Similar reductions in seed weight and subsequent yield traits were reported in sorghum [67], mustard [44], and mungbean [143] under Cd stress. However, the direct correlation with seed Cd accumulation has rarely been addressed.

The compensatory mechanisms underlying the reduction in seed thickness and surface area in amaranth plants exposed to Cd, Zn, and Mn stress, while maintaining total seed weight, remain speculative. Most likely, these morphometric changes result from a reduction in the primary storage component, starch, located in the perisperm. This potential loss could be simultaneously compensated by an increase in denser protein and/or lipid content within the surrounding peripheral embryo. Amaranth seeds, especially those of grain cultivars, are known for their large starchy perisperm and high-protein embryo [13, 56, 129]. This unique seed composition makes such a shift in the allocation of storage compounds between these tissues plausible as a response to HM stress. The stress may alter the plant's ionomic balance and its ability to transport carbohydrates to developing seeds, resulting in less starch stored in the perisperm and, consequently, smaller surface area and thinner seeds. However, in the absence of direct compositional data, these explanations remain hypothetical. On the other hand, plants can flexibly alter protein profiles during seed development, which may help maintain overall seed viability and nutritional quality despite morphological changes. It has been reported that an increase in seed protein content might be a stress adaptation response, such as to heat [63]. Other possible causes of these changes include altered enzyme activities or hormonal signaling, both of which are fundamental during seed development. To confirm these hypotheses, rigorous follow-up biochemical characterization is essential, specifically involving quantitative assessment of total starch and amylose fractions, complemented by proteomic and lipidomic profiling under HM stress. Furthermore, histological analyses and identification of candidate genes regulating seed protein concentration and deposition – similar to the quantitative trait loci in species such as pea—are necessary to elucidate the molecular basis of these changes in plants [165].

### Localization of heavy metals in amaranth seeds

Dithizone staining has been successfully used as a rapid screening method for the histochemical localization of Zn in wheat seeds [100], dehusked brown rice grains [30], and *Moso bamboo* seeds [57]. Its ability to chelate Mn, however, is disputable, though predicted. This dye also interfered with Cd localization, e. g., in leaves of *Arabidopsis* [160], roots of poplar [158], and willow species [130], as well as with Pb in roots of *Cucumis sativus* [150], *Rhus chinensis* [54], and leaves of *Zea mays* [95].

Slightly distinct staining patterns observed in both intact and cross-sectioned seeds in this study likely reflect biological variability in mature seeds, which are otherwise visually indistinguishable. It has been shown that in mature seeds, seed storage proteins and mineral nutrients are concentrated in discrete membrane-bound deposits within the cells, known as protein bodies (PBs), which may vary in size, number, and morphology between differentiated seed tissues, as well as between and within species [13, 19]. Micronutrients, including Zn, form globoid crystal inclusions in PBs and serve the future needs of plant germination until full development [80]. These PBs have previously been histochemically visualized by Fast Green FCF and Aniline Blue Black [13, 19], as general stains mainly recognizing proteins, and by electron microscopy [19, 96]. We assume that dithizone staining of amaranth seeds in this study revealed predominant Zn accumulation in PBs over less abundant elements such as Cd, Pb, and Mn, as the staining pattern was the same across all treatments tested. As we have shown, Zn content in the seeds was relatively high (66.60 ± 6.35 mg kg^−1^ DW), and remained unchanged with Zn-soil supplementation. Moreover, Ozturk et al. [100] observed no staining in seeds containing less than 15 mg kg^−1^ Zn, indicating that other cations, such as Ca^2+^, Mg^2+^, and K^+^, did not interfere with the formation of the red Zn-dithizone complexes. However, their observations were not performed at higher magnifications.

### Ionomic response of amaranth tissues to heavy metal stress

Plant ionome reflects the complex interactions among elements and their dynamic responses to environmental stressors [39]. Exposure to HMs often disrupts nutrient uptake and homeostasis, inhibiting accumulation of various macro- and micronutrients [7, 14, 134].

In *A. cruentus*, most of the analyzed elements (except Zn) were preferentially accumulated in the roots rather than in the above-ground organs. This aligns with the root system's function as a primary barrier that limits translocation of HMs to the shoots [29]. Interestingly, relatively few significant changes in element composition were detected after FDR correction in amaranth tissues after HM use. However, several elements exhibited moderate effects of treatment, indicating subtle but consistent treatment-related trends (Supplementary Table S2). For instance, Cd exposure did not substantially alter nutrient profiles in most organs, however, effect size estimates suggest the presence of moderate, treatment-related responses. This observation contrasts with our previous findings, where Cd markedly inhibited the accumulation of Mg and Mn in roots [60]. Such discrepancies may be attributed to differences in Cd bioavailability between the experimental matrices (hydroponic vs. soil) and the variations in exposure duration [27, 99]. In addition, under Cd application, K content in the roots tended to decrease, though not significantly, consistent with the 22–25% reduction reported by Osmolovskaya et al. [99]. Although Cd and K utilize different transporters, the observed decline may be mediated by the regulatory effects of Cd on K transporter gene expressions and vice versa, as shown in sweet potato studies by Huang et al. [58]. Furthermore, the reduction of Al concentrations in amaranth seeds following Cd, Pb, and Zn exposure, suggests a limited Al translocation to reproductive tissues. Zinc supplementation also influenced nutrient homeostasis, but mostly without significant changes. In the present study, Zn supplementation was associated with decreased root concentrations of Ca, Cu, and Mg. These observations align with previous reports in maize, suggesting that Zn may interfere with the uptake of Mn, Fe, Ca, P, Mg, Ni, Co, and K [14]. A particularly notable finding was the antagonism between Zn and Mn. While Mn supplementation reduced Zn levels in the roots, Zn application caused a non-significant reduction in Mn. However, the moderate effect size suggests a consistent treatment-related trend that may reflect antagonistic interactions between Zn and Mn (Supplementary Table S2). We hypothesize that this interaction could be attributed by similar uptake mechanisms for these divalent cations. Specifically, competition may occur via common transport systems, such as the ZIP (Zinc/Iron-regulated transporter-like protein) and NRAMP (Natural Resistance-Associated Macrophage Protein) transporter families when both ions are present [15, 116]. Furthermore, we cannot rule out the possibility that Zn-Mn antagonism arises from their competition for a limited pool of ligands, such as nicotianamine, or from inherent differences in their mobility within the xylem and phloem sap [21]. While similar antagonistic relationships between Zn and Mn have been documented in other crops, including maize, oilseed rape, and sunflower [38, 66, 115], the precise mechanisms underlying these interactions require further investigation.

Regarding above-ground organs, the impact of HMs was generally limited, although some moderate effect sizes suggest subtle shifts in nutrient homeostasis (Supplementary Table S2). Cadmium and Pb caused minor, non-significant declines in leaf K and Zn content, while Pb exposure additionally led to a slight reduction in Cu levels in both leaves and inflorescences.

Despite relatively slight alterations in elemental profiles across treatments, HMs modulated the patterns of inter-element correlations in amaranth tissues. The consistency of several correlation pairs across treatments suggests that certain ionomic frameworks are conserved under environmental stresses [29]. At the same time, many observed correlations were tissue-specific, a pattern aligning with findings reported in rice exposed to metal toxicity [39].

Ionome analysis of amaranth roots revealed a persistent positive Ca/Mn correlation across Cd, Pb, and Zn treatments. This consistent pattern may suggest a coordinated uptake and/or metabolic regulation of these nutrients under HM stress. Such synergy potentially reflecting shared transport mechanisms or a role in maintaining cellular stability and redox balance [6, 25, 53]. Competitive interactions between Cd and Zn are known due to their similar chemical properties and use of the same transporters [101, 102], although uptake specificity can vary even within plant families [147]. For example, Cd was positively correlated with Ca in basil roots [128], and with K, Na, Mg, and Fe in mulberry [36], illustrating species-dependent ionomic responses. The effects of Cd and Pb were reflected in significant positive Al/Fe, Ba/Zn, and Mo/Ni correlations in amaranth roots, which may be consistent with altered uptake or retention of certain micronutrients and trace elements. Notably, Pb stress generated the most complex correlation network, with more than 30 significant element pairs. Lead displayed direct positive Cd/Cu correlations in roots despite differences in their mobility and translocation [34]. Comparing tissues, under Pb treatment, further demonstrate that roots displayed the most significant correlations, while leaves, inflorescences, and seeds showed fewer elemental associations. These findings confirm that plant roots are the primary site for interacting with, absorbing, and accumulating Pb from the soil [136].

As expected, the ionomes of amaranth leaves and inflorescences were also altered by the presence of HMs, exhibiting several positive and negative correlation pairs. Homologous elements in leaves commonly show significant correlations [146]. Cadmium modified the correlation patterns among elements such as Cu, Fe, K, and Zn in amaranth leaves, while Mn caused ten shifts in leaves, both positive and negative. In contrast, Pb had a more limited influence, affecting only three correlation pairs. Zinc excess showed a positive correlation with Cu, and, together with the decrease in Zn in leaves, this suggests the use of shared transport pathways [51]. Furthermore, Zn treatment produced Ca/Sr correlation in leaves, a relationship previously observed in *Achillea millefolium*, *Alchemilla xanthochlora*, *Phleum rhaeticum,* and *Ranunculus acris* [47] as well as in various vegetable species [147]. These findings indicate that the essential element Ca and the non-essential Sr share the same transport system [146].

Seeds of Cd- and Mn-treated plants exhibited the most striking shifts. Application of Cd produced 35 significant positive correlations compared with only 5 in control, with Ca, Fe, K, Mg, Na, and Zn especially affected. Cadmium itself did not correlate with any of these elements, and their concentrations remained relative to controls. This pattern is consistant with potential indirect influence of Cd onthe seed ionome, despite its concentration in seeds being significantly elevated. Possible mechanisms for Cd indirect and seed-specific effects likely involve transporter regulation, chelation, or nutrient partitioning [12, 17]. This interpretation aligns with findings in sorghum grains, where Cd accumulation did not correlate with Zn, Mn, Fe, or Ca, despite clear physiological impacts [144]. Similarly, Mn was associated with shifts in ionomic relationships in seeds, even though its own concentration did not increase significantly. In seeds of Mn-treated plants, positive correlations among Mn/K, Fe, Ba, Al, and Ca suggest stress-induced shifts in nutrient regulation. Comparable complexity has been reported in cereals, where Zn/Cd interactions can be synergistic and antagonistic, depending on tissue type and growth stage [104]. Based on ionomic analysis, our findings indicate that HM exposure is associated with changes in the amaranth ionome that appear to involve alterations in nutrient coordination rather than fluctuations in absolute concentrations. Ionomic profiling complemented health risk assessment by revealing tissue-specific metal partitioning. While the substantial Cd accumulation in seeds contrasted with the negligible Pb translocation, Zn and Mn showed stable homeostasis, thus enabling a more precise evaluation of human dietary risk. Given that the present study relies on correlation analyses, the observed relationships should not be interpreted as evidence of direct causal interactions between elements.

Finally, clustering analysis clearly separated roots, leaves, inflorescences, and seeds into four distinct groups, reflecting tissue-specific ionomic characteristics. These results aligned with those of Hunková et al. [60], who demonstrated that amaranth tissues exhibited distinct ionomic responses to Cd, Zn, and Mn under hydroponic conditions. Similar patterns of tissue-based ionomic clustering were reported in mulberry under metal stress [36]. The relatively low contribution of amaranth seeds and inflorescences in multivariate analysis may reflect homeostatic regulation and protection of reproductive tissues. Overall, in the HCA analysis, Cd formed a distinct cluster, indicating a different distribution pattern compared to other elements. This can be explained by the distinct physiological behavior of Cd compared to essential elements and the lack of homeostatic regulation [167].

Despite the comprehensive ionomics and statistical analyses performed, certain limitations of this study need to be acknowledged. The relatively low number of biological replicates may limit statistical power and increase variability in the results. In particular, PCA and correlation network analyses are sensitive to sample size and are therefore presented as exploratory tools to identify general trends rather than definitive relationships. Future studies using nature-contaminated soil with larger sample sizes would be valuable for validating and strengthening the robustness of the observed ionomic interactions and multivariate patterns.

## Conclusions

Amaranth, a promising pseudocereal, has considerable potential to contribute to sustainable food systems under climate change conditions. In this study, we evaluated the bioaccumulation and translocation of Cd, Pb, Zn, and Mn in the Slovak grain *A. cruentus* cv. Pribina grown under semi-field conditions, as well as the associated health risks to consumers. Our findings demonstrated that consuming amaranth seeds from Cd-contaminated soils may pose a significant carcinogenic risk to humans, particularly adults, while Pb, Zn, and Mn concentrations in seeds did not exceed health concern thresholds. Morphometric analysis showed that mainly Cd, Mn, and Zn contamination primarily affected several seed morphometric traits. Moreover, the metals were specifically localized within the seed protein bodies. Ionomic profiling further revealed that roots and leaves act as central regulators of nutrient homeostasis under HM stress. The observed tissue-specific patterns provide new insights into the uptake, translocation, accumulation, and cross-talk of elements in *A. cruentus* exposed to Cd, Pb, Zn, and Mn. Overall, only a limited number of significant nutrient shifts were detected in amaranth plants exposed to soil HMs, even under high contamination. However, the amaranth ionome network was reorganized, revealing conserved nutrient relationships, organ-specific responses, and substantial indirect effects of Cd and Mn on seed nutrient coordination. These findings suggest that *A. cruentus* cv. Pribina possesses adaptive mechanisms that maintain ionomic balance under excessive HMs, likely established early in development. Future research should aim to elucidate the molecular and physiological defense mechanisms underlying this tolerance and to validate these findings in field trials.

## Supplementary Information

Supplementary Material 1.

## Data Availability

All data are included in this article and its supplementary information file.

## Abbreviations

ADI: Average daily intake
BCF: Bioconcentration factor
Cd: Cadmium
CF: Contamination factor
CR: Carcinogenic risk
HCA: Hierarchical cluster analysis
HMs: Heavy metals
ICP-OES: Inductively coupled plasma optical emission spectrophotometry
LOD: Limit of detection
Mn: Manganese
NCR: Non-carcinogenic risk
Pb: Lead
PCA: Principal component analysis
R*f*D: Reference dose
THQ: Target hazard quotient
TF: Translocation factor
Zn: Zinc
