# Supplementary material for: Health risk assessment and ionomic profiling under cadmium, lead, zinc, and manganese stress in Amaranthus cruentus
Source: BMC Plant Biol. 2026 May 20;26:1185. doi: 10.1186/s12870-026-08983-z (PMC13359888; doi:10.1186/s12870-026-08983-z)
Supplement: Supplementary file 1 — Supplementary Material 1. [file 12870_2026_8983_MOESM1_ESM.docx]

**Supplementary material**

**Health risk assessment and ionomic profiling under cadmium, lead, zinc, and manganese stress in *Amaranthus cruentus***

Monika Szabóová, Veronika Lancíková, Jana Kačírová, Andrea Hricová^*^, Veronika Mistríková

Institute of Plant Genetics and Biotechnology, Plant Science and Biodiversity Centre, Slovak Academy of Sciences, Nitra, Slovakia

*Correspondence: Andrea Hricová, Institute of Plant Genetics and Biotechnology, Plant Science and Biodiversity Centre, Slovak Academy of Sciences, Nitra, Slovakia; e-mail: andrea.hricova@savba.sk


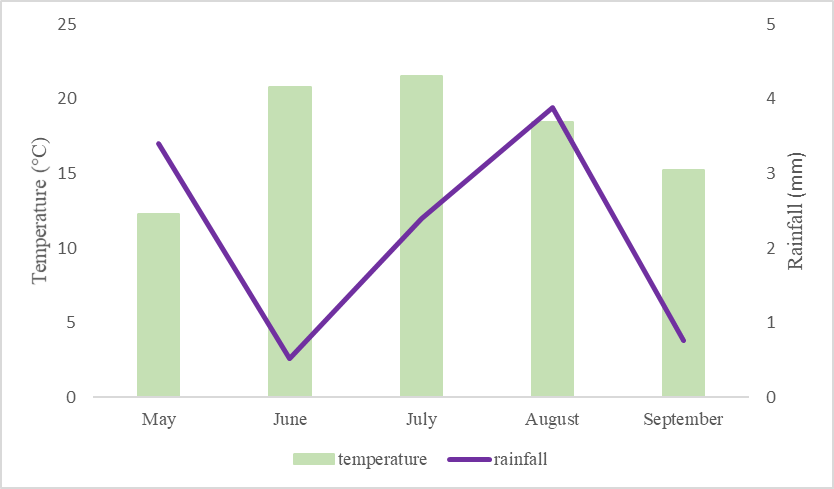


**Figure S1**. Average meteorological conditions during the amaranth cropping season (May 2021– September 2021) in locality Nitra, Slovakia.


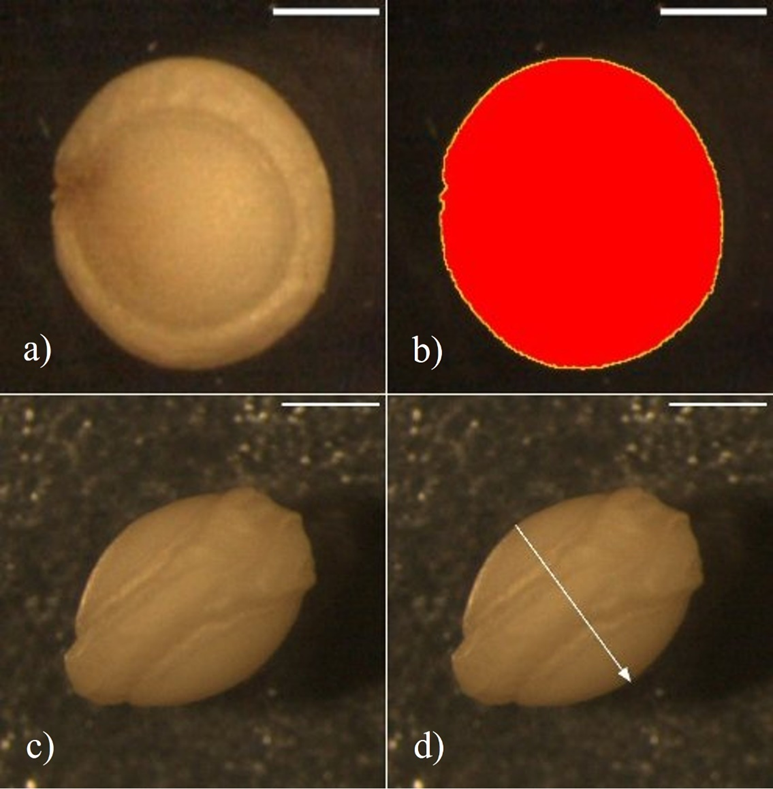


**Figure S2.** Representative images of amaranth (*A. cruentus*) seeds viewed from dorsal (a) and lateral (c) sides as used for measurements of seed area (b) and thickness parameters (d). Bar represents 0.5 mm.

**Figure S3**. The graph shows the proportion of variance explained by each principal component (PC). The black line represents the percentage of variance explained by individual PCs, while the gray bars indicate the cumulative variance explained. The first two principal components (PC1 and PC1) account for the majority of the total variance and were therefore used for further PCA interpretation.

**Table S1**

Concentrations of Cd, Pb, Zn, and Mn and calculated contamination factor in the control and contaminated soil samples used for cultivation of *A. cruentus* plants in a pot until full seed maturity.

| **Metal concentration Treatment** | Cd (mg kg^-1^ DW) | Pb (mg kg^-1^ DW) | | Zn (mg kg^-1^ DW) | Mn (mg kg^-1^ DW) | CF |
| --- | --- | --- | --- | --- | --- | --- |
| **Control** | 0.82 ± 0.13 | | 13.70 ± 1.74 | 116.40 ± 10.08 | 742.18 ± 69.38 | 1.00 ± 0.00 |
| **Cd** | 93.96 ± 13.52* | | 13.96 ± 0.60 | 122.76 ± 1.25 | 714.80 ± 58.22 | 108. 35 ± 12.77* |
| **Pb** | 1.00 ± 0.03 | | 1147.26 ± 449.89* | 118.66 ± 5.10 | 1089.26 ± 285.70 | 81.91 ± 22.12* |
| **Zn** | 0.81 ± 0.24 | | 16.99 ± 2.19 | 321.32 ± 35.14* | 629.12 ± 68.62 | 2.78 ± 0.48 |
| **Mn** | 0.85 ± 0.09 | | 12.53 ± 1.55 | 115.99 ± 29.68 | 1933.31 ± 720.45* | 2.20 ± 0.76 |

Data are means of three biological replicates ± standard deviations (n = 3). Statistically significant differences compared to the control at *p* < 0.05 are marked with *. Abbreviations: contamination factor (CF), dry weight (DW).

**Table S2**. Effect sizes (Cohen's *f*) and FDR-adjusted *p*-values from one-way ANOVA evaluating HM-treatment effects on elemental composition in amaranth tissues

|  | **Root** | | **Leaf** | | **Inflorescence** | | **Seed** | |
| --- | --- | --- | --- | --- | --- | --- | --- | --- |
|  | *p* | *f* | *p* | *f* | *p* | *f* | *p* | *f* |
| **Al** | 0.282 | 0.767 | 0.821 | 0.387 | 0.650 | 0.504 | 0.076 | 1.080 |
| **Ba** | 0.442 | 0.639 | 0.519 | 0.587 | 0.641 | 0.510 | 0.443 | 0.638 |
| **Ca** | 0.335 | 0.7204 | 0.865 | 0.352 | 0.601 | 0.535 | 0.481 | 0.612 |
| **Cd** | <0.0001 | 4.353 | <0.0001 | 7.025 | 0.001 | 2.113 | 0.094 | 1.034 |
| **Cr** | 0.323 | 0.7307 | 0.452 | 0.000 | 0.452 | 0.000 | 0.452 | 0.000 |
| **Cu** | 0.436 | 0.6434 | 0.087 | 1.052 | 0.168 | 0.899 | 0.356 | 0.703 |
| **Fe** | 0.469 | 0.621 | 0.546 | 0.570 | 0.636 | 0.512 | 0.329 | 0.726 |
| **K** | 0.637 | 0.512 | 0.423 | 0.653 | 0.097 | 1.029 | 0.358 | 0.701 |
| **Mg** | 0.297 | 0.754 | 0.986 | 0.183 | 0.355 | 0.704 | 0.068 | 1.111 |
| **Mn** | 0.055 | 1.158 | 0.327 | 0.727 | 0.520 | 0.586 | 0.342 | 0.714 |
| **Mo** | 0.645 | 0.507 | 0.029 | 1.307 | 0.475 | 0.617 | 0.452 | 0.633 |
| **Na** | 0.138 | 0.946 | 0.638 | 0.511 | 0.777 | 0.420 | 0.959 | 0.244 |
| **Ni** | 0.515 | 0.590 | 0.012 | 1.504 | 0.072 | 1.095 | 0.003 | 1.845 |
| **Pb** | 0.0002 | 2.663 | 0.452 | 0.000 | 0.452 | 0.000 | 0.452 | 0.000 |
| **Sr** | 0.366 | 0.695 | 0.631 | 0.515 | 0.335 | 0.720 | 0.454 | 0.631 |
| **Zn** | 0.497 | 1.368 | 0.293 | 0.757 | 0.483 | 0.611 | 0.620 | 0.523 |

Abbreviations: Cohen' s *f* (*f*), FDR-adjusted *p*-value (*p*).

**Table S3**. Correlations among elements in the amaranth root tissues estimated by Pearson correlation coefficients. Statistically significant differences at p < 0.05 are marked with *.

1. Control

|  | Al | Ba | Ca | Cd | Cr | Cu | Fe | K | Mg | Mn | Mo | Na | Ni | Pb | Sr | Zn |
| --- | --- | --- | --- | --- | --- | --- | --- | --- | --- | --- | --- | --- | --- | --- | --- | --- |
| Al | 1.000 |  |  |  |  |  |  |  |  |  |  |  |  |  |  |  |
| Ba | 0.906 | 1.000 |  |  |  |  |  |  |  |  |  |  |  |  |  |  |
| Ca | 0.791 | 0.976 | 1.000 |  |  |  |  |  |  |  |  |  |  |  |  |  |
| Cd | 0.528 | 0.838 | 0.937 | 1.000 |  |  |  |  |  |  |  |  |  |  |  |  |
| Cr | 0.978 | 0.974 | 0.901 | 0.692 | 1.000 |  |  |  |  |  |  |  |  |  |  |  |
| Cu | 0.980 | 0.972 | 0.897 | 0.687 | 1.000 | 1.000 |  |  |  |  |  |  |  |  |  |  |
| Fe | 0.973 | 0.979 | 0.911 | 0.710 | 1.000 | 0.999 | 1.000 |  |  |  |  |  |  |  |  |  |
| K | -0.891 | -0.999 | -0.983 | -0.856 | -0.966 | -0.964 | -0.972 | 1.000 |  |  |  |  |  |  |  |  |
| Mg | 0.987 | 0.962 | 0.880 | 0.658 | 0.999 | 0.999 | 0.997 | -0.952 | 1.000 |  |  |  |  |  |  |  |
| Mn | 0.962 | 0.987 | 0.929 | 0.741 | 0.998 | 0.997 | 0.999 | -0.981 | 0.993 | 1.000 |  |  |  |  |  |  |
| Mo | 0.993 | 0.949 | 0.858 | 0.624 | 0.996 | 0.997 | 0.993 | -0.938 | 0.999 | 0.987 | 1.000 |  |  |  |  |  |
| Na | -0.998 | -0.876 | -0.749 | -0.471 | -0.963 | -0.965 | -0.956 | 0.859 | -0.974 | -0.941 | -0.983 | 1.000 |  |  |  |  |
| Ni | 0.997 | 0.938 | 0.839 | 0.596 | 0.992 | 0.993 | 0.989 | -0.925 | 0.997 | 0.981 | 0.999 | -0.989 | 1.000 |  |  |  |
| Pb | 0.974 | 0.978 | 0.909 | 0.707 | 1.000 | 1.000 | 1.000 | -0.971 | 0.998 | 0.999 | 0.994 | -0.957 | 0.989 | 1.000 |  |  |
| Sr | 0.770 | 0.968 | 0.999 | 0.948 | 0.885 | 0.882 | 0.896 | -0.976 | 0.863 | 0.916 | 0.840 | -0.726 | 0.820 | 0.894 | 1.000 |  |
| Zn | 0.753 | 0.961 | 0.998 | 0.957 | 0.872 | 0.869 | 0.884 | -0.970 | 0.849 | 0.904 | 0.825 | -0.707 | 0.804 | 0.882 | 1.000 | 1.000 |

1. Cadmium treatment

|  | Al | Ba | Ca | Cd | Cr | Cu | Fe | K | Mg | Mn | Mo | Na | Ni | Pb | Sr | Zn |
| --- | --- | --- | --- | --- | --- | --- | --- | --- | --- | --- | --- | --- | --- | --- | --- | --- |
| Al | 1.000 |  |  |  |  |  |  |  |  |  |  |  |  |  |  |  |
| Ba | 0.986 | 1.000 |  |  |  |  |  |  |  |  |  |  |  |  |  |  |
| Ca | 0.864 | 0.769 | 1.000 |  |  |  |  |  |  |  |  |  |  |  |  |  |
| Cd | -0.262 | -0.417 | 0.260 | 1.000 |  |  |  |  |  |  |  |  |  |  |  |  |
| Cr | 0.982 | 0.938 | 0.943 | -0.076 | 1.000 |  |  |  |  |  |  |  |  |  |  |  |
| Cu | 0.984 | 0.942 | 0.939 | -0.088 | 1.000* | 1.000 |  |  |  |  |  |  |  |  |  |  |
| Fe | 0.999* | 0.979 | 0.882 | -0.225 | 0.989 | 0.990 | 1.000 |  |  |  |  |  |  |  |  |  |
| K | -0.947 | -0.881 | -0.980 | -0.062 | -0.990 | -0.989 | -0.958 | 1.000 |  |  |  |  |  |  |  |  |
| Mg | 0.809 | 0.702 | 0.995 | 0.355 | 0.905 | 0.900 | 0.831 | -0.955 | 1.000 |  |  |  |  |  |  |  |
| Mn | 0.889 | 0.801 | 0.999* | 0.210 | 0.959 | 0.955 | 0.905 | -0.989 | 0.989 | 1.000 |  |  |  |  |  |  |
| Mo | 0.999* | 0.979 | 0.883 | -0.224 | 0.989 | 0.990 | 1.000* | -0.959 | 0.831 | 0.906 | 1.000 |  |  |  |  |  |
| Na | -0.033 | 0.132 | -0.532 | -0.956 | -0.219 | -0.208 | -0.071 | 0.352 | -0.613 | -0.487 | -0.071 | 1.000 |  |  |  |  |
| Ni | 0.999* | 0.977 | 0.887 | -0.215 | 0.990 | 0.992 | 1.000* | -0.961 | 0.837 | 0.910 | 1.000* | -0.081 | 1.000 |  |  |  |
| Pb | 0.963 | 0.906 | 0.967 | 0.006 | 0.997 | 0.996 | 0.973 | -0.998* | 0.937 | 0.979 | 0.973 | -0.299 | 0.975 | 1.000 |  |  |
| Sr | 0.893 | 0.807 | 0.998* | 0.199 | 0.962 | 0.959 | 0.910 | -0.990 | 0.987 | 1.000* | 0.910 | -0.478 | 0.914 | 0.981 | 1.000 |  |
| Zn | 0.977 | 0.999* | 0.735 | -0.463 | 0.919 | 0.924 | 0.968 | -0.856 | 0.664 | 0.769 | 0.968 | 0.183 | 0.965 | 0.883 | 0.776 | 1.000 |

1. Lead treatment

|  | Al | Ba | Ca | Cd | Cr | Cu | Fe | K | Mg | Mn | Mo | Na | Ni | Pb | Sr | Zn |
| --- | --- | --- | --- | --- | --- | --- | --- | --- | --- | --- | --- | --- | --- | --- | --- | --- |
| Al | 1.000 |  |  |  |  |  |  |  |  |  |  |  |  |  |  |  |
| Ba | 0.995 | 1.000 |  |  |  |  |  |  |  |  |  |  |  |  |  |  |
| Ca | 0.990 | 0.999* | 1.000 |  |  |  |  |  |  |  |  |  |  |  |  |  |
| Cd | 0.993 | 0.976 | 0.968 | 1.000 |  |  |  |  |  |  |  |  |  |  |  |  |
| Cr | 0.993 | 1.000* | 1.000* | 0.973 | 1.000 |  |  |  |  |  |  |  |  |  |  |  |
| Cu | 0.996 | 0.981 | 0.974 | 1.000* | 0.978 | 1.000 |  |  |  |  |  |  |  |  |  |  |
| Fe | 1.000* | 0.997* | 0.994 | 0.990 | 0.996 | 0.993 | 1.000 |  |  |  |  |  |  |  |  |  |
| K | -0.995 | -1.000* | -0.999* | -0.976 | -1.000* | -0.981 | -0.997 | 1.000 |  |  |  |  |  |  |  |  |
| Mg | 0.448 | 0.536 | 0.567 | 0.342 | 0.551 | 0.364 | 0.470 | -0.538 | 1.000 |  |  |  |  |  |  |  |
| Mn | 0.991 | 0.999* | 1.000* | 0.969 | 1.000* | 0.974 | 0.994 | -1.000* | 0.565 | 1.000 |  |  |  |  |  |  |
| Mo | 0.979 | 0.994 | 0.998* | 0.948 | 0.996 | 0.956 | 0.983 | -0.995 | 0.622 | 0.997* | 1.000 |  |  |  |  |  |
| Na | -0.998* | -0.999* | -0.997* | -0.984 | -0.998* | -0.988 | -0.999* | 0.999* | -0.503 | -0.997* | -0.990 | 1.000 |  |  |  |  |
| Ni | 0.982 | 0.996 | 0.999* | 0.953 | 0.997* | 0.960 | 0.986 | -0.996 | 0.610 | 0.998* | 1.000* | -0.992 | 1.000 |  |  |  |
| Pb | 0.992 | 0.974 | 0.966 | 1.000* | 0.970 | 0.999* | 0.989 | -0.974 | 0.333 | 0.966 | 0.945 | -0.982 | 0.950 | 1.000 |  |  |
| Sr | 1.000* | 0.996 | 0.993 | 0.991 | 0.995 | 0.994 | 1.000* | -0.996 | 0.462 | 0.993 | 0.982 | -0.999* | 0.985 | 0.990 | 1.000 |  |
| Zn | 0.999* | 0.999* | 0.996 | 0.987 | 0.997* | 0.990 | 1.000* | -0.998* | 0.490 | 0.996 | 0.987 | -1.000* | 0.990 | 0.985 | 0.999* | 1.000 |

1. Zinc treatment

|  | Al | Ba | Ca | Cd | Cr | Cu | Fe | K | Mg | Mn | Mo | Na | Ni | Pb | Sr | Zn |
| --- | --- | --- | --- | --- | --- | --- | --- | --- | --- | --- | --- | --- | --- | --- | --- | --- |
| Al | 1.000 |  |  |  |  |  |  |  |  |  |  |  |  |  |  |  |
| Ba | 0.998* | 1.000 |  |  |  |  |  |  |  |  |  |  |  |  |  |  |
| Ca | 1.000* | 0.999 | 1.000 |  |  |  |  |  |  |  |  |  |  |  |  |  |
| Cd | 0.796 | 0.830 | 0.807 | 1.000 |  |  |  |  |  |  |  |  |  |  |  |  |
| Cr | 0.620 | 0.664 | 0.634 | 0.968 | 1.000 |  |  |  |  |  |  |  |  |  |  |  |
| Cu | 0.956 | 0.971 | 0.961 | 0.939 | 0.823 | 1.000 |  |  |  |  |  |  |  |  |  |  |
| Fe | 0.923 | 0.944 | 0.930 | 0.967 | 0.874 | 0.995 | 1.000 |  |  |  |  |  |  |  |  |  |
| K | 0.251 | 0.194 | 0.233 | -0.386 | -0.604 | -0.045 | -0.140 | 1.000 |  |  |  |  |  |  |  |  |
| Mg | 0.967 | 0.951 | 0.963 | 0.617 | 0.401 | 0.850 | 0.796 | 0.488 | 1.000 |  |  |  |  |  |  |  |
| Mn | 0.999* | 1.000* | 1.000* | 0.821 | 0.652 | 0.967 | 0.939 | 0.210 | 0.956 | 1.000 |  |  |  |  |  |  |
| Mo | 0.836 | 0.867 | 0.846 | 0.998* | 0.949 | 0.960 | 0.983 | -0.322 | 0.670 | 0.858 | 1.000 |  |  |  |  |  |
| Na | -0.105 | -0.162 | -0.123 | -0.685 | -0.845 | -0.392 | -0.479 | 0.937 | 0.151 | -0.146 | -0.633 | 1.000 |  |  |  |  |
| Ni | 0.946 | 0.964 | 0.952 | 0.949 | 0.840 | 1.000* | 0.998* | -0.076 | 0.834 | 0.959 | 0.969 | -0.421 | 1.000 |  |  |  |
| Pb | -0.470 | -0.418 | -0.454 | 0.160 | 0.401 | -0.191 | -0.095 | -0.972 | -0.679 | -0.433 | 0.091 | -0.828 | -0.160 | 1.000 |  |  |
| Sr | 0.994 | 0.987 | 0.992 | 0.728 | 0.534 | 0.920 | 0.878 | 0.351 | 0.989 | 0.989 | 0.774 | 0.000 | 0.907 | -0.560 | 1.000 |  |
| Zn | 0.953 | 0.933 | 0.947 | 0.574 | 0.352 | 0.821 | 0.763 | 0.533 | 0.999* | 0.939 | 0.630 | 0.203 | 0.803 | -0.716 | 0.979 | 1.000 |

1. Manganese treatment

|  | Al | Ba | Ca | Cd | Cr | Cu | Fe | K | Mg | Mn | Mo | Na | Ni | Pb | Sr | Zn |
| --- | --- | --- | --- | --- | --- | --- | --- | --- | --- | --- | --- | --- | --- | --- | --- | --- |
| Al | 1.000 |  |  |  |  |  |  |  |  |  |  |  |  |  |  |  |
| Ba | 0.887 | 1.000 |  |  |  |  |  |  |  |  |  |  |  |  |  |  |
| Ca | 0.154 | -0.320 | 1.000 |  |  |  |  |  |  |  |  |  |  |  |  |  |
| Cd | 0.976 | 0.966 | -0.065 | 1.000 |  |  |  |  |  |  |  |  |  |  |  |  |
| Cr | 0.933 | 0.994 | -0.213 | 0.989 | 1.000 |  |  |  |  |  |  |  |  |  |  |  |
| Cu | 0.896 | 1.000* | -0.300 | 0.971 | 0.996 | 1.000 |  |  |  |  |  |  |  |  |  |  |
| Fe | 0.988 | 0.947 | 0.002 | 0.998* | 0.977 | 0.953 | 1.000 |  |  |  |  |  |  |  |  |  |
| K | 0.163 | 0.601 | -0.950 | 0.374 | 0.508 | 0.584 | 0.311 | 1.000 |  |  |  |  |  |  |  |  |
| Mg | 0.113 | -0.359 | 0.999* | -0.106 | -0.253 | -0.339 | -0.039 | -0.962 | 1.000 |  |  |  |  |  |  |  |
| Mn | 0.763 | 0.378 | 0.756 | 0.603 | 0.478 | 0.397 | 0.656 | -0.513 | 0.729 | 1.000 |  |  |  |  |  |  |
| Mo | 0.984 | 0.955 | -0.026 | 0.999* | 0.982 | 0.961 | 1.000* | 0.338 | -0.067 | 0.634 | 1.000 |  |  |  |  |  |
| Na | 0.904 | 0.604 | 0.562 | 0.789 | 0.688 | 0.620 | 0.828 | -0.275 | 0.528 | 0.966 | 0.812 | 1.000 |  |  |  |  |
| Ni | 0.919 | 0.997* | -0.249 | 0.983 | 0.999* | 0.999* | 0.968 | 0.540 | -0.289 | 0.445 | 0.975 | 0.661 | 1.000 |  |  |  |
| Pb | 0.938 | 0.672 | 0.486 | 0.840 | 0.750 | 0.688 | 0.875 | -0.188 | 0.450 | 0.939 | 0.861 | 0.996 | 0.725 | 1.000 |  |  |
| Sr | 0.991 | 0.940 | 0.023 | 0.996 | 0.972 | 0.947 | 1.000* | 0.291 | -0.018 | 0.672 | 0.999 | 0.840 | 0.962 | 0.885 | 1.000 |  |
| Zn | 0.825 | 0.471 | 0.685 | 0.683 | 0.566 | 0.490 | 0.730 | -0.422 | 0.654 | 0.995 | 0.711 | 0.988 | 0.535 | 0.970 | 0.745 | 1.000 |

**Table S4**. Correlations among elements in the amaranth leaf tissues estimated by Pearson correlation coefficients. Statistically significant differences at p < 0.05 are marked with *.

1. Control

|  | Al | Ba | Ca | Cd | Cu | Fe | K | Mg | Mn | Mo | Na | Ni | Pb | Sr | Zn |
| --- | --- | --- | --- | --- | --- | --- | --- | --- | --- | --- | --- | --- | --- | --- | --- |
| Al | 1.000 |  |  |  |  |  |  |  |  |  |  |  |  |  |  |
| Ba | -0.945 | 1.000 |  |  |  |  |  |  |  |  |  |  |  |  |  |
| Ca | -0.998* | 0.964 | 1.000 |  |  |  |  |  |  |  |  |  |  |  |  |
| Cd | 0.344 | -0.018 | -0.282 | 1.000 |  |  |  |  |  |  |  |  |  |  |  |
| Cu | 0.846 | -0.974 | -0.879 | -0.210 | 1.000 |  |  |  |  |  |  |  |  |  |  |
| Fe | 0.384 | -0.664 | -0.443 | -0.735 | 0.817 | 1.000 |  |  |  |  |  |  |  |  |  |
| K | 0.175 | -0.486 | -0.238 | -0.865 | 0.672 | 0.976 | 1.000 |  |  |  |  |  |  |  |  |
| Mg | -0.932 | 0.999* | 0.954 | 0.019 | -0.982 | -0.692 | -0.519 | 1.000 |  |  |  |  |  |  |  |
| Mn | -0.478 | 0.739 | 0.534 | 0.660 | -0.873 | -0.995 | -0.948 | 0.764 | 1.000 |  |  |  |  |  |  |
| Mo | -0.405 | 0.681 | 0.464 | 0.719 | -0.830 | -1.000* | -0.971 | 0.709 | 0.997 | 1.000 |  |  |  |  |  |
| Na | -0.974 | 0.847 | 0.958 | -0.546 | -0.704 | -0.166 | 0.052 | 0.827 | 0.268 | 0.189 | 1.000 |  |  |  |  |
| Ni | -0.331 | 0.005 | 0.270 | -1.000* | 0.223 | 0.744 | 0.871 | -0.033 | -0.670 | -0.728 | 0.535 | 1.000 |  |  |  |
| Pb | 0.481 | -0.168 | -0.423 | 0.989 | -0.060 | -0.625 | -0.779 | -0.131 | 0.540 | 0.607 | -0.666 | -0.987 | 1.000 |  |  |
| Sr | -0.903 | 0.994 | 0.929 | 0.092 | -0.993 | -0.743 | -0.580 | 0.997* | 0.809 | 0.758 | 0.784 | -0.105 | -0.059 | 1.000 |  |
| Zn | -0.301 | -0.026 | 0.239 | -0.999 | 0.253 | 0.765 | 0.886 | -0.064 | -0.693 | -0.750 | 0.508 | 0.999 | -0.981 | -0.137 | 1.000 |

1. Cadmium treatment

|  | Al | Ba | Ca | Cd | Cu | Fe | K | Mg | Mn | Mo | Na | Ni | Pb | Sr | Zn |
| --- | --- | --- | --- | --- | --- | --- | --- | --- | --- | --- | --- | --- | --- | --- | --- |
| Al | 1.000 |  |  |  |  |  |  |  |  |  |  |  |  |  |  |
| Ba | -0.992 | 1.000 |  |  |  |  |  |  |  |  |  |  |  |  |  |
| Ca | -0.974 | 0.937 | 1.000 |  |  |  |  |  |  |  |  |  |  |  |  |
| Cd | 0.293 | -0.413 | -0.069 | 1.000 |  |  |  |  |  |  |  |  |  |  |  |
| Cu | 0.391 | -0.505 | -0.173 | 0.995 | 1.000 |  |  |  |  |  |  |  |  |  |  |
| Fe | 0.371 | -0.487 | -0.152 | 0.997 | 1.000* | 1.000 |  |  |  |  |  |  |  |  |  |
| K | -0.991 | 1.000* | 0.936 | -0.416 | -0.508 | -0.490 | 1.000 |  |  |  |  |  |  |  |  |
| Mg | 0.942 | -0.891 | -0.993 | -0.046 | 0.058 | 0.037 | -0.889 | 1.000 |  |  |  |  |  |  |  |
| Mn | -0.625 | 0.720 | 0.433 | -0.929 | -0.963 | -0.957 | 0.722 | -0.326 | 1.000 |  |  |  |  |  |  |
| Mo | 0.525 | -0.630 | -0.320 | 0.967 | 0.988 | 0.985 | -0.633 | 0.208 | -0.993 | 1.000 |  |  |  |  |  |
| Na | 0.928 | -0.968 | -0.820 | 0.628 | 0.705 | 0.690 | -0.969 | 0.749 | -0.871 | 0.804 | 1.000 |  |  |  |  |
| Ni | 0.909 | -0.848 | -0.979 | -0.133 | -0.029 | -0.050 | -0.846 | 0.996 | -0.242 | 0.122 | 0.688 | 1.000 |  |  |  |
| Pb | 0.166 | -0.038 | -0.384 | -0.894 | -0.843 | -0.854 | -0.034 | 0.488 | 0.666 | -0.752 | -0.213 | 0.562 | 1.000 |  |  |
| Sr | -0.987 | 0.999* | 0.924 | -0.445 | -0.536 | -0.518 | 0.999* | -0.874 | 0.745 | -0.658 | -0.977 | -0.828 | -0.002 | 1.000 |  |
| Zn | -0.998* | 0.997* | 0.960 | -0.347 | -0.442 | -0.424 | 0.997* | -0.921 | 0.668 | -0.573 | -0.948 | -0.883 | -0.109 | 0.994 | 1.000 |

1. Lead treatment

|  | Al | Ba | Ca | Cd | Cu | Fe | K | Mg | Mn | Mo | Na | Ni | Pb | Sr | Zn |
| --- | --- | --- | --- | --- | --- | --- | --- | --- | --- | --- | --- | --- | --- | --- | --- |
| Al | 1.000 |  |  |  |  |  |  |  |  |  |  |  |  |  |  |
| Ba | -0.609 | 1.000 |  |  |  |  |  |  |  |  |  |  |  |  |  |
| Ca | -0.624 | 1.000* | 1.000 |  |  |  |  |  |  |  |  |  |  |  |  |
| Cd | -0.857 | 0.931 | 0.938 | 1.000 |  |  |  |  |  |  |  |  |  |  |  |
| Cu | -0.924 | 0.259 | 0.276 | 0.594 | 1.000 |  |  |  |  |  |  |  |  |  |  |
| Fe | 0.979 | -0.436 | -0.452 | -0.734 | -0.982 | 1.000 |  |  |  |  |  |  |  |  |  |
| K | 0.768 | 0.040 | 0.022 | -0.327 | -0.955 | 0.882 | 1.000 |  |  |  |  |  |  |  |  |
| Mg | -0.477 | 0.987 | 0.984 | 0.862 | 0.103 | -0.288 | 0.198 | 1.000 |  |  |  |  |  |  |  |
| Mn | -0.983 | 0.745 | 0.757 | 0.937 | 0.837 | -0.925 | -0.637 | 0.630 | 1.000 |  |  |  |  |  |  |
| Mo | -0.983 | 0.451 | 0.467 | 0.746 | 0.979 | -1.000* | -0.873 | 0.305 | 0.932 | 1.000 |  |  |  |  |  |
| Na | -0.051 | -0.761 | -0.749 | -0.471 | 0.430 | -0.253 | -0.679 | -0.854 | -0.133 | 0.236 | 1.000 |  |  |  |  |
| Ni | 0.181 | -0.890 | -0.882 | -0.663 | 0.209 | -0.022 | -0.491 | -0.951 | -0.359 | 0.005 | 0.973 | 1.000 |  |  |  |
| Pb | -0.510 | 0.993 | 0.990 | 0.880 | 0.141 | -0.324 | 0.160 | 0.999 | 0.659 | 0.341 | -0.833 | -0.939 | 1.000 |  |  |
| Sr | -0.700 | 0.993 | 0.995 | 0.968 | 0.373 | -0.540 | -0.080 | 0.961 | 0.819 | 0.555 | -0.677 | -0.829 | 0.971 | 1.000 |  |
| Zn | -0.987 | 0.729 | 0.741 | 0.928 | 0.850 | -0.934 | -0.655 | 0.612 | 1.000* | 0.940 | -0.110 | -0.337 | 0.641 | 0.806 | 1.000 |

1. Zinc treatment

|  | Al | Ba | Ca | Cd | Cu | Fe | K | Mg | Mn | Mo | Na | Ni | Pb | Sr | Zn |
| --- | --- | --- | --- | --- | --- | --- | --- | --- | --- | --- | --- | --- | --- | --- | --- |
| Al | 1.000 |  |  |  |  |  |  |  |  |  |  |  |  |  |  |
| Ba | -0.511 | 1.000 |  |  |  |  |  |  |  |  |  |  |  |  |  |
| Ca | -0.350 | 0.984 | 1.000 |  |  |  |  |  |  |  |  |  |  |  |  |
| Cd | -0.430 | 0.996 | 0.996 | 1.000 |  |  |  |  |  |  |  |  |  |  |  |
| Cu | -0.146 | 0.925 | 0.978 | 0.956 | 1.000 |  |  |  |  |  |  |  |  |  |  |
| Fe | 0.973 | -0.297 | -0.122 | -0.208 | 0.087 | 1.000 |  |  |  |  |  |  |  |  |  |
| K | -0.925 | 0.147 | -0.031 | 0.056 | -0.239 | -0.988 | 1.000 |  |  |  |  |  |  |  |  |
| Mg | 0.328 | 0.644 | 0.770 | 0.712 | 0.886 | 0.539 | -0.662 | 1.000 |  |  |  |  |  |  |  |
| Mn | -0.344 | 0.983 | 1.000* | 0.996 | 0.979 | -0.116 | -0.038 | 0.774 | 1.000 |  |  |  |  |  |  |
| Mo | 0.978 | -0.320 | -0.146 | -0.231 | 0.064 | 1.000* | -0.984 | 0.518 | -0.140 | 1.000 |  |  |  |  |  |
| Na | -0.953 | 0.225 | 0.048 | 0.135 | -0.161 | -0.997* | 0.997 | -0.600 | 0.042 | -0.995 | 1.000 |  |  |  |  |
| Ni | -0.498 | -0.492 | -0.639 | -0.569 | -0.785 | -0.685 | 0.789 | -0.983 | -0.643 | -0.668 | 0.738 | 1.000 |  |  |  |
| Pb | 0.680 | 0.283 | 0.449 | 0.370 | 0.626 | 0.832 | -0.907 | 0.916 | 0.455 | 0.818 | -0.871 | -0.974 | 1.000 |  |  |
| Sr | -0.385 | 0.990 | 0.999* | 0.999* | 0.969 | -0.160 | 0.007 | 0.745 | 0.999* | -0.184 | 0.086 | -0.609 | 0.415 | 1.000 |  |
| Zn | -0.107 | 0.909 | 0.969 | 0.944 | 0.999* | 0.127 | -0.278 | 0.904 | 0.970 | 0.104 | -0.201 | -0.809 | 0.656 | 0.959 | 1.000 |

1. Manganese treatment

|  | Al | Ba | Ca | Cd | Cu | Fe | K | Mg | Mn | Mo | Na | Ni | Pb | Sr | Zn |
| --- | --- | --- | --- | --- | --- | --- | --- | --- | --- | --- | --- | --- | --- | --- | --- |
| Al | 1.000 |  |  |  |  |  |  |  |  |  |  |  |  |  |  |
| Ba | -0.895 | 1.000 |  |  |  |  |  |  |  |  |  |  |  |  |  |
| Ca | -0.992 | 0.945 | 1.000 |  |  |  |  |  |  |  |  |  |  |  |  |
| Cd | -0.903 | 1.000* | 0.951 | 1.000 |  |  |  |  |  |  |  |  |  |  |  |
| Cu | 0.564 | -0.136 | -0.452 | -0.154 | 1.000 |  |  |  |  |  |  |  |  |  |  |
| Fe | 0.997* | -0.860 | -0.980 | -0.869 | 0.622 | 1.000 |  |  |  |  |  |  |  |  |  |
| K | 0.456 | -0.805 | -0.567 | -0.795 | -0.478 | 0.390 | 1.000 |  |  |  |  |  |  |  |  |
| Mg | -0.555 | 0.125 | 0.443 | 0.143 | -1.000* | -0.614 | 0.487 | 1.000 |  |  |  |  |  |  |  |
| Mn | -0.994 | 0.842 | 0.972 | 0.852 | -0.648 | -0.999* | -0.359 | 0.640 | 1.000 |  |  |  |  |  |  |
| Mo | -0.955 | 0.987 | 0.985 | 0.990 | -0.293 | -0.931 | -0.700 | 0.283 | 0.918 | 1.000 |  |  |  |  |  |
| Na | -0.507 | 0.839 | 0.614 | 0.829 | 0.426 | -0.443 | -0.998* | -0.436 | 0.413 | 0.740 | 1.000 |  |  |  |  |
| Ni | 0.935 | -0.679 | -0.882 | -0.692 | 0.820 | 0.959 | 0.112 | -0.813 | -0.968 | -0.788 | -0.169 | 1.000 |  |  |  |
| Pb | -0.734 | 0.354 | 0.641 | 0.371 | -0.975 | -0.782 | 0.269 | 0.972 | 0.803 | 0.500 | -0.212 | -0.927 | 1.000 |  |  |
| Sr | -0.904 | 1.000* | 0.952 | 1.000* | -0.157 | -0.871 | -0.792 | 0.146 | 0.854 | 0.990 | 0.827 | -0.695 | 0.375 | 1.000 |  |
| Zn | -0.933 | 0.996 | 0.972 | 0.997* | -0.229 | -0.905 | -0.746 | 0.218 | 0.890 | 0.998* | 0.783 | -0.745 | 0.441 | 0.997* | 1.000 |

**Table S5**. Correlations among elements in the amaranth inflorescence tissues estimated by Pearson correlation coefficients. Statistically significant differences at p < 0.05 are marked with *.

1. Control

|  | Al | Ba | Ca | Cd | Cu | Fe | K | Mg | Mn | Mo | Na | Ni | Pb | Sr | Zn |
| --- | --- | --- | --- | --- | --- | --- | --- | --- | --- | --- | --- | --- | --- | --- | --- |
| Al | 1.000 |  |  |  |  |  |  |  |  |  |  |  |  |  |  |
| Ba | 0.694 | 1.000 |  |  |  |  |  |  |  |  |  |  |  |  |  |
| Ca | -0.357 | 0.425 | 1.000 |  |  |  |  |  |  |  |  |  |  |  |  |
| Cd | 0.014 | -0.711 | -0.939 | 1.000 |  |  |  |  |  |  |  |  |  |  |  |
| Cu | 0.794 | 0.989 | 0.284 | -0.597 | 1.000 |  |  |  |  |  |  |  |  |  |  |
| Fe | 0.893 | 0.943 | 0.100 | -0.437 | 0.982 | 1.000 |  |  |  |  |  |  |  |  |  |
| K | 0.759 | 0.058 | -0.879 | 0.661 | 0.207 | 0.386 | 1.000 |  |  |  |  |  |  |  |  |
| Mg | -0.062 | -0.762 | -0.910 | 0.997* | -0.656 | -0.504 | 0.603 | 1.000 |  |  |  |  |  |  |  |
| Mn | -0.275 | 0.502 | 0.996 | -0.965 | 0.366 | 0.186 | -0.835 | -0.943 | 1.000 |  |  |  |  |  |  |
| Mo | 0.859 | 0.965 | 0.171 | -0.500 | 0.993 | 0.997* | 0.319 | -0.564 | 0.256 | 1.000 |  |  |  |  |  |
| Na | 0.061 | 0.761 | 0.910 | -0.997* | 0.655 | 0.503 | -0.603 | -1.000* | 0.943 | 0.563 | 1.000 |  |  |  |  |
| Ni | 0.920 | 0.356 | -0.694 | 0.404 | 0.493 | 0.646 | 0.953 | 0.334 | -0.629 | 0.590 | -0.335 | 1.000 |  |  |  |
| Pb | 0.014 | -0.711 | -0.939 | 1.000* | -0.597 | -0.437 | 0.661 | 0.997* | -0.965 | -0.500 | -0.997* | 0.404 | 1.000 |  |  |
| Sr | 0.949 | 0.886 | -0.043 | -0.304 | 0.946 | 0.990 | 0.514 | -0.375 | 0.044 | 0.977 | 0.374 | 0.749 | -0.304 | 1.000 |  |
| Zn | -0.218 | 0.551 | 0.989 | -0.979 | 0.420 | 0.243 | -0.801 | -0.960 | 0.998* | 0.312 | 0.961 | -0.583 | -0.979 | 0.102 | 1.000 |

1. Cadmium treatment

|  | Al | Ba | Ca | Cd | Cu | Fe | K | Mg | Mn | Mo | Na | Ni | Pb | Sr | Zn |
| --- | --- | --- | --- | --- | --- | --- | --- | --- | --- | --- | --- | --- | --- | --- | --- |
| Al | 1.000 |  |  |  |  |  |  |  |  |  |  |  |  |  |  |
| Ba | 0.987 | 1.000 |  |  |  |  |  |  |  |  |  |  |  |  |  |
| Ca | 0.956 | 0.991 | 1.000 |  |  |  |  |  |  |  |  |  |  |  |  |
| Cd | 0.839 | 0.916 | 0.962 | 1.000 |  |  |  |  |  |  |  |  |  |  |  |
| Cu | 0.983 | 1.000* | 0.993 | 0.924 | 1.000 |  |  |  |  |  |  |  |  |  |  |
| Fe | 0.988 | 1.000* | 0.990 | 0.914 | 1.000* | 1.000 |  |  |  |  |  |  |  |  |  |
| K | 0.867 | 0.936 | 0.975 | 0.999* | 0.943 | 0.934 | 1.000 |  |  |  |  |  |  |  |  |
| Mg | -0.653 | -0.522 | -0.402 | -0.136 | -0.504 | -0.526 | -0.189 | 1.000 |  |  |  |  |  |  |  |
| Mn | 0.998* | 0.995 | 0.973 | 0.873 | 0.993 | 0.996 | 0.898 | -0.602 | 1.000 |  |  |  |  |  |  |
| Mo | 0.850 | 0.924 | 0.967 | 1.000* | 0.932 | 0.922 | 0.999* | -0.157 | 0.883 | 1.000 |  |  |  |  |  |
| Na | 0.772 | 0.865 | 0.924 | 0.994 | 0.875 | 0.862 | 0.986 | -0.023 | 0.812 | 0.991 | 1.000 |  |  |  |  |
| Ni | 1.000* | 0.989 | 0.959 | 0.846 | 0.985 | 0.989 | 0.873 | -0.644 | 0.999* | 0.857 | 0.780 | 1.000 |  |  |  |
| Pb | 0.334 | 0.176 | 0.042 | -0.233 | 0.156 | 0.182 | -0.181 | -0.932 | 0.271 | -0.212 | -0.342 | 0.322 | 1.000 |  |  |
| Sr | 0.888 | 0.951 | 0.984 | 0.995 | 0.957 | 0.949 | 0.999* | -0.231 | 0.916 | 0.997* | 0.978 | 0.893 | -0.138 | 1.000 |  |
| Zn | 0.966 | 0.995 | 0.999* | 0.951 | 0.997* | 0.995 | 0.966 | -0.436 | 0.981 | 0.957 | 0.910 | 0.969 | 0.079 | 0.976 | 1.000 |

1. Lead treatment

|  | Al | Ba | Ca | Cd | Cu | Fe | K | Mg | Mn | Mo | Na | Ni | Pb | Sr | Zn |
| --- | --- | --- | --- | --- | --- | --- | --- | --- | --- | --- | --- | --- | --- | --- | --- |
| Al | 1.000 |  |  |  |  |  |  |  |  |  |  |  |  |  |  |
| Ba | -0.288 | 1.000 |  |  |  |  |  |  |  |  |  |  |  |  |  |
| Ca | -0.928 | 0.625 | 1.000 |  |  |  |  |  |  |  |  |  |  |  |  |
| Cd | -0.021 | 0.963 | 0.392 | 1.000 |  |  |  |  |  |  |  |  |  |  |  |
| Cu | -0.793 | 0.812 | 0.963 | 0.626 | 1.000 |  |  |  |  |  |  |  |  |  |  |
| Fe | 0.947 | -0.581 | -0.998* | -0.341 | -0.946 | 1.000 |  |  |  |  |  |  |  |  |  |
| K | 0.653 | 0.536 | -0.324 | 0.743 | -0.056 | 0.376 | 1.000 |  |  |  |  |  |  |  |  |
| Mg | 0.364 | 0.787 | 0.009 | 0.924 | 0.279 | 0.046 | 0.943 | 1.000 |  |  |  |  |  |  |  |
| Mn | -0.929 | 0.622 | 1.000* | 0.389 | 0.962 | -0.999* | -0.327 | 0.006 | 1.000 |  |  |  |  |  |  |
| Mo | -0.894 | -0.171 | 0.663 | -0.429 | 0.436 | -0.703 | -0.923 | -0.743 | 0.665 | 1.000 |  |  |  |  |  |
| Na | -0.750 | -0.417 | 0.449 | -0.646 | 0.191 | -0.498 | -0.991 | -0.889 | 0.452 | 0.967 | 1.000 |  |  |  |  |
| Ni | -0.855 | 0.743 | 0.987 | 0.535 | 0.994 | -0.976 | -0.167 | 0.171 | 0.986 | 0.533 | 0.299 | 1.000 |  |  |  |
| Pb | 0.876 | -0.714 | -0.993 | -0.500 | -0.988 | 0.985 | 0.208 | -0.130 | -0.992 | -0.568 | -0.338 | -0.999 | 1.000 |  |  |
| Sr | -0.681 | 0.898 | 0.905 | 0.746 | 0.986 | -0.880 | 0.110 | 0.434 | 0.904 | 0.281 | 0.026 | 0.962 | -0.950 | 1.000 |  |
| Zn | -0.945 | 0.586 | 0.999* | 0.346 | 0.948 | -1.000* | -0.370 | -0.040 | 0.999* | 0.699 | 0.493 | 0.978 | -0.986 | 0.883 | 1.000 |

1. Zinc treatment

|  | Al | Ba | Ca | Cd | Cu | Fe | K | Mg | Mn | Mo | Na | Ni | Pb | Sr | Zn |
| --- | --- | --- | --- | --- | --- | --- | --- | --- | --- | --- | --- | --- | --- | --- | --- |
| Al | 1.000 |  |  |  |  |  |  |  |  |  |  |  |  |  |  |
| Ba | 0.398 | 1.000 |  |  |  |  |  |  |  |  |  |  |  |  |  |
| Ca | -0.778 | 0.267 | 1.000 |  |  |  |  |  |  |  |  |  |  |  |  |
| Cd | 0.305 | 0.995 | 0.360 | 1.000 |  |  |  |  |  |  |  |  |  |  |  |
| Cu | -0.973 | -0.174 | 0.903 | -0.076 | 1.000 |  |  |  |  |  |  |  |  |  |  |
| Fe | 0.994 | 0.299 | -0.840 | 0.203 | -0.992 | 1.000 |  |  |  |  |  |  |  |  |  |
| K | -0.850 | 0.145 | 0.992 | 0.241 | 0.949 | -0.901 | 1.000 |  |  |  |  |  |  |  |  |
| Mg | 0.395 | 1.000* | 0.269 | 0.995 | -0.171 | 0.296 | 0.147 | 1.000 |  |  |  |  |  |  |  |
| Mn | -0.741 | -0.911 | 0.155 | -0.866 | 0.565 | -0.666 | 0.276 | -0.910 | 1.000 |  |  |  |  |  |  |
| Mo | 0.977 | 0.583 | -0.628 | 0.500 | -0.902 | 0.950 | -0.720 | 0.581 | -0.866 | 1.000 |  |  |  |  |  |
| Na | 0.972 | 0.603 | -0.608 | 0.521 | -0.891 | 0.942 | -0.702 | 0.601 | -0.878 | 1.000* | 1.000 |  |  |  |  |
| Ni | 0.997 | 0.466 | -0.729 | 0.377 | -0.952 | 0.984 | -0.808 | 0.464 | -0.789 | 0.991 | 0.987 | 1.000 |  |  |  |
| Pb | -0.977 | -0.583 | 0.628 | -0.500 | 0.902 | -0.950 | 0.720 | -0.581 | 0.866 | -1.000* | -1.000* | -0.991 | 1.000 |  |  |
| Sr | -0.204 | 0.817 | 0.773 | 0.870 | 0.425 | -0.306 | 0.688 | 0.819 | -0.507 | 0.008 | 0.033 | -0.129 | -0.008 | 1.000 |  |
| Zn | -0.949 | -0.088 | 0.937 | 0.010 | 0.996 | -0.977 | 0.973 | -0.085 | 0.491 | -0.861 | -0.848 | -0.922 | 0.861 | 0.502 | 1.000 |

1. Manganese treatment

|  | Al | Ba | Ca | Cd | Cu | Fe | K | Mg | Mn | Mo | Na | Ni | Pb | Sr | Zn |
| --- | --- | --- | --- | --- | --- | --- | --- | --- | --- | --- | --- | --- | --- | --- | --- |
| Al | 1.000 |  |  |  |  |  |  |  |  |  |  |  |  |  |  |
| Ba | 0.981 | 1.000 |  |  |  |  |  |  |  |  |  |  |  |  |  |
| Ca | 0.998* | 0.966 | 1.000 |  |  |  |  |  |  |  |  |  |  |  |  |
| Cd | 0.982 | 1.000* | 0.967 | 1.000 |  |  |  |  |  |  |  |  |  |  |  |
| Cu | 0.948 | 0.992 | 0.925 | 0.991 | 1.000 |  |  |  |  |  |  |  |  |  |  |
| Fe | 0.997 | 0.993 | 0.989 | 0.994 | 0.970 | 1.000 |  |  |  |  |  |  |  |  |  |
| K | -0.235 | -0.042 | -0.298 | -0.046 | 0.088 | -0.157 | 1.000 |  |  |  |  |  |  |  |  |
| Mg | -0.225 | -0.409 | -0.160 | -0.405 | -0.524 | -0.301 | -0.895 | 1.000 |  |  |  |  |  |  |  |
| Mn | 0.996 | 0.959 | 1.000* | 0.960 | 0.914 | 0.985 | -0.325 | -0.132 | 1.000 |  |  |  |  |  |  |
| Mo | 0.982 | 1.000* | 0.967 | 1.000* | 0.991 | 0.994 | -0.046 | -0.405 | 0.960 | 1.000 |  |  |  |  |  |
| Na | 0.972 | 0.999* | 0.954 | 0.999* | 0.996 | 0.987 | 0.002 | -0.448 | 0.945 | 0.999* | 1.000 |  |  |  |  |
| Ni | 0.976 | 1.000* | 0.959 | 1.000* | 0.995 | 0.990 | -0.017 | -0.432 | 0.951 | 1.000* | 1.000* | 1.000 |  |  |  |
| Pb | 0.327 | 0.504 | 0.264 | 0.500 | 0.612 | 0.401 | 0.842 | -0.994 | 0.237 | 0.500 | 0.541 | 0.525 | 1.000 |  |  |
| Sr | 0.994 | 0.996 | 0.985 | 0.997 | 0.977 | 1.000* | -0.127 | -0.330 | 0.979 | 0.997 | 0.992 | 0.994 | 0.428 | 1.000 |  |
| Zn | 0.983 | 1.000* | 0.969 | 1.000* | 0.990 | 0.995 | -0.053 | -0.399 | 0.962 | 1.000* | 0.999* | 0.999 | 0.494 | 0.997* | 1.000 |

**Table S6**. Correlations among elements in the amaranth seed tissues estimated by Pearson correlation coefficients. Statistically significant differences at p < 0.05 are marked with *.

1. Control

|  | Al | Ba | Ca | Cd | Cu | Fe | K | Mg | Mn | Mo | Na | Ni | Pb | Sr | Zn |
| --- | --- | --- | --- | --- | --- | --- | --- | --- | --- | --- | --- | --- | --- | --- | --- |
| Al | 1.000 |  |  |  |  |  |  |  |  |  |  |  |  |  |  |
| Ba | -0.981 | 1.000 |  |  |  |  |  |  |  |  |  |  |  |  |  |
| Ca | -0.960 | 0.887 | 1.000 |  |  |  |  |  |  |  |  |  |  |  |  |
| Cd | -0.645 | 0.482 | 0.832 | 1.000 |  |  |  |  |  |  |  |  |  |  |  |
| Cu | -0.266 | 0.072 | 0.524 | 0.909 | 1.000 |  |  |  |  |  |  |  |  |  |  |
| Fe | -0.414 | 0.227 | 0.651 | 0.963 | 0.988 | 1.000 |  |  |  |  |  |  |  |  |  |
| K | -0.991 | 0.944 | 0.990 | 0.743 | 0.396 | 0.535 | 1.000 |  |  |  |  |  |  |  |  |
| Mg | -0.787 | 0.892 | 0.583 | 0.035 | -0.386 | -0.237 | 0.694 | 1.000 |  |  |  |  |  |  |  |
| Mn | -0.176 | -0.021 | 0.443 | 0.866 | 0.996 | 0.969 | 0.309 | -0.470 | 1.000 |  |  |  |  |  |  |
| Mo | 0.984 | -1.000* | -0.896 | -0.500 | -0.093 | -0.247 | -0.951 | -0.883 | 0.000 | 1.000 |  |  |  |  |  |
| Na | -0.145 | 0.336 | -0.137 | -0.663 | -0.915 | -0.841 | 0.008 | 0.725 | -0.949 | -0.316 | 1.000 |  |  |  |  |
| Ni | 0.789 | -0.653 | -0.929 | -0.978 | -0.802 | -0.886 | -0.866 | -0.241 | -0.744 | 0.669 | 0.494 | 1.000 |  |  |  |
| Pb | -0.645 | 0.482 | 0.832 | 1.000* | 0.909 | 0.963 | 0.743 | 0.035 | 0.866 | -0.500 | -0.663 | -0.978 | 1.000 |  |  |
| Sr | -0.790 | 0.895 | 0.587 | 0.040 | -0.381 | -0.232 | 0.698 | 1.000* | -0.465 | -0.885 | 0.721 | -0.246 | 0.040 | 1.000 |  |
| Zn | -0.591 | 0.421 | 0.792 | 0.998* | 0.935 | 0.979 | 0.696 | -0.034 | 0.898 | -0.439 | -0.713 | -0.962 | 0.998* | -0.029 | 1.000 |

1. Cadmium treatment

|  | Al | Ba | Ca | Cd | Cu | Fe | K | Mg | Mn | Mo | Na | Ni | Pb | Sr | Zn |
| --- | --- | --- | --- | --- | --- | --- | --- | --- | --- | --- | --- | --- | --- | --- | --- |
| Al | 1.000 |  |  |  |  |  |  |  |  |  |  |  |  |  |  |
| Ba | 0.998* | 1.000 |  |  |  |  |  |  |  |  |  |  |  |  |  |
| Ca | 1.000* | 0.999* | 1.000 |  |  |  |  |  |  |  |  |  |  |  |  |
| Cd | 0.559 | 0.612 | 0.582 | 1.000 |  |  |  |  |  |  |  |  |  |  |  |
| Cu | 0.953* | 0.970 | 0.961 | 0.785 | 1.000 |  |  |  |  |  |  |  |  |  |  |
| Fe | 1.000* | 0.999* | 1.000* | 0.571 | 0.957 | 1.000 |  |  |  |  |  |  |  |  |  |
| K | 1.000* | 0.999* | 1.000* | 0.575 | 0.958 | 1.000* | 1.000 |  |  |  |  |  |  |  |  |
| Mg | 1.000 | 0.999* | 1.000* | 0.574 | 0.958 | 1.000* | 1.000* | 1.000 |  |  |  |  |  |  |  |
| Mn | 0.952 | 0.970 | 0.960 | 0.786 | 1.000* | 0.956 | 0.958 | 0.957 | 1.000 |  |  |  |  |  |  |
| Mo | 0.855 | 0.888 | 0.869 | 0.908 | 0.973 | 0.863 | 0.866 | 0.865 | 0.973 | 1.000 |  |  |  |  |  |
| Na | 1.000* | 0.999* | 1.000* | 0.576 | 0.959 | 1.000* | 1.000* | 1.000 | 0.958 | 0.866 | 1.000 |  |  |  |  |
| Ni | 0.992 | 0.981 | 0.988 | 0.447 | 0.905 | 0.990 | 0.989 | 0.989 | 0.905 | 0.782 | 0.989 | 1.000 |  |  |  |
| Pb | 0.876 | 0.843 | 0.863 | 0.090 | 0.688 | 0.869 | 0.866 | 0.867 | 0.687 | 0.500 | 0.866 | 0.931 | 1.000 |  |  |
| Sr | 1.000* | 0.999* | 1.000* | 0.574 | 0.958 | 1.000* | 1.000* | 1.000* | 0.958 | 0.865 | 1.000* | 0.989 | 0.867 | 1.000 |  |
| Zn | 1.000* | 0.997 | 0.999* | 0.546 | 0.948 | 1.000* | 0.999* | 0.999* | 0.947 | 0.848 | 0.999* | 0.993 | 0.883 | 0.999* | 1.000 |

1. Lead treatment

|  | Al | Ba | Ca | Cd | Cu | Fe | K | Mg | Mn | Mo | Na | Ni | Pb | Sr | Zn |
| --- | --- | --- | --- | --- | --- | --- | --- | --- | --- | --- | --- | --- | --- | --- | --- |
| Al | 1.000 |  |  |  |  |  |  |  |  |  |  |  |  |  |  |
| Ba | -0.909 | 1.000 |  |  |  |  |  |  |  |  |  |  |  |  |  |
| Ca | -0.132 | 0.532 | 1.000 |  |  |  |  |  |  |  |  |  |  |  |  |
| Cd | -0.842 | 0.990 | 0.646 | 1.000 |  |  |  |  |  |  |  |  |  |  |  |
| Cu | -0.958 | 0.990 | 0.410 | 0.961 | 1.000 |  |  |  |  |  |  |  |  |  |  |
| Fe | 0.883 | -0.607 | 0.350 | -0.489 | -0.711 | 1.000 |  |  |  |  |  |  |  |  |  |
| K | 0.335 | 0.088 | 0.890 | 0.227 | -0.051 | 0.739 | 1.000 |  |  |  |  |  |  |  |  |
| Mg | -0.267 | 0.644 | 0.990 | 0.745 | 0.532 | 0.218 | 0.819 | 1.000 |  |  |  |  |  |  |  |
| Mn | 0.148 | 0.277 | 0.961 | 0.410 | 0.142 | 0.595 | 0.981 | 0.914 | 1.000 |  |  |  |  |  |  |
| Mo | 0.888 | -0.617 | 0.338 | -0.500 | -0.720 | 1.000* | 0.730 | 0.205 | 0.585 | 1.000 |  |  |  |  |  |
| Na | -0.994 | 0.857 | 0.021 | 0.777 | 0.920 | -0.929 | -0.437 | 0.158 | -0.257 | -0.934 | 1.000 |  |  |  |  |
| Ni | -0.024 | 0.438 | 0.994 | 0.560 | 0.309 | 0.449 | 0.934 | 0.970 | 0.985 | 0.438 | -0.087 | 1.000 |  |  |  |
| Pb | -0.842 | 0.990 | 0.646 | 1.000* | 0.961 | -0.489 | 0.227 | 0.745 | 0.410 | -0.500 | 0.777 | 0.560 | 1.000 |  |  |
| Sr | -0.861 | 0.995 | 0.618 | 0.999* | 0.971 | -0.520 | 0.191 | 0.720 | 0.376 | -0.531 | 0.799 | 0.529 | 0.999* | 1.000 |  |
| Zn | 0.077 | 0.345 | 0.978 | 0.473 | 0.212 | 0.537 | 0.965 | 0.940 | 0.997* | 0.526 | -0.187 | 0.995 | 0.473 | 0.441 | 1.000 |

1. Zinc treatment

|  | Al | Ba | Ca | Cd | Cu | Fe | K | Mg | Mn | Mo | Na | Ni | Pb | Sr | Zn |
| --- | --- | --- | --- | --- | --- | --- | --- | --- | --- | --- | --- | --- | --- | --- | --- |
| Al | 1.000 |  |  |  |  |  |  |  |  |  |  |  |  |  |  |
| Ba | 0.716 | 1.000 |  |  |  |  |  |  |  |  |  |  |  |  |  |
| Ca | 1.000* | 0.711 | 1.000 |  |  |  |  |  |  |  |  |  |  |  |  |
| Cd | 0.794 | 0.993 | 0.790 | 1.000 |  |  |  |  |  |  |  |  |  |  |  |
| Cu | -0.595 | 0.136 | -0.601 | 0.016 | 1.000 |  |  |  |  |  |  |  |  |  |  |
| Fe | -0.998* | -0.758 | -0.998* | -0.830 | 0.544 | 1.000 |  |  |  |  |  |  |  |  |  |
| K | 0.966 | 0.511 | 0.968 | 0.610 | -0.782 | -0.948 | 1.000 |  |  |  |  |  |  |  |  |
| Mg | 0.907 | 0.355 | 0.910 | 0.464 | -0.878 | -0.879 | 0.985 | 1.000 |  |  |  |  |  |  |  |
| Mn | 0.729 | 1.000* | 0.724 | 0.995 | 0.117 | -0.770 | 0.527 | 0.372 | 1.000 |  |  |  |  |  |  |
| Mo | -0.923 | -0.393 | -0.926 | -0.500 | 0.858 | 0.898 | -0.991 | -0.999* | -0.410 | 1.000 |  |  |  |  |  |
| Na | 0.920 | 0.932 | 0.917 | 0.969 | -0.232 | -0.942 | 0.787 | 0.669 | 0.939 | -0.699 | 1.000 |  |  |  |  |
| Ni | 0.129 | -0.600 | 0.136 | -0.500 | -0.874 | -0.067 | 0.381 | 0.535 | -0.585 | -0.500 | -0.270 | 1.000 |  |  |  |
| Pb | 0.794 | 0.993 | 0.790 | 1.000* | 0.016 | -0.830 | 0.610 | 0.464 | 0.995 | -0.500 | 0.969 | -0.500 | 1.000 |  |  |
| Sr | 0.935 | 0.917 | 0.933 | 0.958 | -0.272 | -0.955 | 0.812 | 0.699 | 0.924 | -0.728 | 0.999* | -0.230 | 0.958 | 1.000 |  |
| Zn | -0.496 | 0.251 | -0.503 | 0.133 | 0.993 | 0.441 | -0.704 | -0.816 | 0.233 | 0.792 | -0.116 | -0.925 | 0.133 | -0.157 | 1.000 |

1. Manganese treatment

|  | Al | Ba | Ca | Cd | Cu | Fe | K | Mg | Mn | Mo | Na | Ni | Pb | Sr | Zn |
| --- | --- | --- | --- | --- | --- | --- | --- | --- | --- | --- | --- | --- | --- | --- | --- |
| Al | 1.000 |  |  |  |  |  |  |  |  |  |  |  |  |  |  |
| Ba | 1.000* | 1.000 |  |  |  |  |  |  |  |  |  |  |  |  |  |
| Ca | 1.000* | 0.999* | 1.000 |  |  |  |  |  |  |  |  |  |  |  |  |
| Cd | 0.556 | 0.565 | 0.534 | 1.000 |  |  |  |  |  |  |  |  |  |  |  |
| Cu | 0.968 | 0.971 | 0.961 | 0.746 | 1.000 |  |  |  |  |  |  |  |  |  |  |
| Fe | 1.000* | 1.000* | 1.000* | 0.558 | 0.969 | 1.000 |  |  |  |  |  |  |  |  |  |
| K | 0.998* | 0.997 | 0.999* | 0.497 | 0.948 | 0.997* | 1.000 |  |  |  |  |  |  |  |  |
| Mg | 0.999* | 1.000* | 0.998* | 0.587 | 0.977 | 0.999* | 0.994 | 1.000 |  |  |  |  |  |  |  |
| Mn | 0.998* | 0.997* | 0.999* | 0.500 | 0.950 | 0.998* | 1.000* | 0.995 | 1.000 |  |  |  |  |  |  |
| Mo | 0.441 | 0.432 | 0.465 | -0.500 | 0.203 | 0.439 | 0.503 | 0.407 | 0.500 | 1.000 |  |  |  |  |  |
| Na | -0.022 | -0.011 | -0.049 | 0.819 | 0.229 | -0.020 | -0.092 | 0.016 | -0.088 | -0.907 | 1.000 |  |  |  |  |
| Ni | 0.430 | 0.420 | 0.454 | -0.511 | 0.190 | 0.428 | 0.492 | 0.395 | 0.489 | 1.000* | -0.912 | 1.000 |  |  |  |
| Pb | 0.556 | 0.565 | 0.534 | 1.000* | 0.746 | 0.558 | 0.497 | 0.587 | 0.500 | -0.500 | 0.819 | -0.511 | 1.000 |  |  |
| Sr | 0.997* | 0.998* | 0.995 | 0.615 | 0.984 | 0.998 | 0.990 | 0.999* | 0.990 | 0.375 | 0.050 | 0.363 | 0.615 | 1.000 |  |
| Zn | 0.991 | 0.990 | 0.995 | 0.442 | 0.927 | 0.991 | 0.998* | 0.986 | 0.998 | 0.555 | -0.153 | 0.545 | 0.442 | 0.979 | 1.000 |

**Table S7**. Quantitative parameters of correlation networks

| Tissue | Treatment | Path length | Network density | Clustering coefficient |
| --- | --- | --- | --- | --- |
| Root | Cd | 1.000 | 0.078 | 0.269 |
|  | Pb | 1.210 | 0.181 | 0.415 |
|  | Zn | 1.000 | 0.082 | 0.152 |
|  | Mn | 1.103 | 0.100 | 0.233 |
| Leaf | Control | 1.182 | 0.069 | 0.000 |
|  | Cd | 1.000 | 0.167 | 0.190 |
|  | Pb | 1.000 | 0.100 | 0.000 |
|  | Zn | 1.000 | 0.097 | 0.130 |
|  | Mn | 1.130 | 0.076 | 0.111 |
| Inflorescence | Control | 1.000 | 0.100 | 0.200 |
|  | Cd | 1.000 | 0.1430 | 0.25 |
|  | Pb | 1.000 | 0.500 | 0.500 |
|  | Zn | 1.000 | 0.200 | 0.300 |
|  | Mn | 1.054 | 0.164 | 0.236 |
| Seed | Control | 1.000 | 0.119 | 0.214 |
|  | Cd | 1.109 | 0.265 | 0.333 |
|  | Pb | 1.000 | 0.119 | 0.214 |
|  | Zn | 1.000 | 0.064 | 0.136 |
|  | Mn | 1.297 | 0.154 | 0.242 |
